# Bioinformatic analysis of wheat defensin gene family and function verification of candidate genes

**DOI:** 10.3389/fpls.2023.1279502

**Published:** 2023-10-24

**Authors:** Ye Dong, Youning Wang, Mingshuang Tang, Wang Chen, Yi Chai, Wenli Wang

**Affiliations:** ^1^ Ministry of Agriculture and Rural Affairs (MARA) Key Laboratory of Sustainable Crop Production in the Middle Reaches of the Yangtze River (Co-Construction by Ministry and Province), College of Agriculture, Yangtze University, Jingzhou, China; ^2^ Hubei Key Laboratory of Quality Control of Characteristic Fruits and Vegetables, Hubei Engineering University, Xiaogan, Hubei, China; ^3^ Nanchong Academy of Agriculture Sciences, Nanchong, Sichuan, China; ^4^ College of Plant Protection, Northwest A&F University, Yangling, Shanxi, China

**Keywords:** defensins, gene structure, subcellular localization, gene expression, *Phytophthora infestans* infection

## Abstract

Plant defensins are widely distributed in the leaves, fruits, roots, stems, seeds, and tubers. Research shows that defensin in plants play a significant role in physiological metabolism, growth and development. Plant defensins can kill and suppress a variety of pathogenic bacteria. In this study, we understand the phylogenetic relationships, protein characterization, chromosomal localization, promoter and gene structural features of the *TaPDFs* family through sequence alignment and conserved protein structural domain analysis. A total of 73 *PDF* gene members in wheat, 15 *PDF* genes in maize, and 11 *PDF* genes in rice were identified. A total of 35, 65, and 34 *PDF* gene members were identified in the genomes of *Ae. tauschii*, *T. urartu*, and *T. dicoccoides*, respectively. *TaPDF4.9* and *TaPDF2.15* were constructed into pART27 vector with YFP by homologous recombination for subcellular localization analysis. Subcellular localization results showed that *TaPDF4.9* and *TaPDF2.15* were basically located in the cell membrane and cytoplasm, and *TaPDF4.9* was also located in the nucleus. *TaPDF4.9* and *TaPDF2.15* could inhibit the infection of *Phytophthora infestans* strain ‘88069’. The results suggest that *TaPDFs* may be able to improve disease resistance. The study of wheat defensins will be beneficial for improving wheat yield and provides a theoretical basis for research on resistance to wheat diseases.

## Introduction

Plants have their own defense systems, and when they are infected with external pathogens such as fungi and oomycetes, they will produce a series of antagonistic substances themselves (Sathoff et al., 2019). Antagonistic substances are mainly categorized into some low molecular weight secondary metabolites or some low molecular weight short peptides less than 100 amino acids to prevent the propagation and expansion of the pathogen and further invasion of the host plant (Terras et al., 1992). Plant defenses are among the short, low molecular weight peptides that are widely distributed in plants. (De Coninck et al., 2013).

There are 45-54 amino acid residues in plant defensins. The encoded proteins were approximately 5 kDa, which contain eight conserved cysteine residues forming four pairs of disulfide bonds (Cys4-Cys7, Cys3-Cys6, Cys2-Cys5 and Cys1-Cys8), among which, the most conserved are the two pairs, Cys2-Cys5 and Cys1-Cys8 (Thomma et al., 2002). It was shown that the solution structure of radish defensin (Rs-AFP1) by nuclear magnetic resonance (NMR) consists of three reverse parallel β-folded lamellae and one α-helix, forming a stable βαβββ structure with four pairs of disulfide bonds acting as support, and the Csαβ modal sequence (Cysteine stabilized αβ motif) was found, fixing a pair of disulfide bonds on the lamellae in the α-helix and β-fold (Vriens et al., 2016). The β-folded lamellar signature sequence (Cys-Xaa-Cys) and the α-helical signature sequence (Cys-Xaa-Xaa-Xaa-Cys) are common to most plant defensins (Parisi et al., 2019). The Cysteine stabilized αβ motif indicates that plant defensins are more structurally stable and can be stably expressed in many plants. Defensins have a special resistance mechanism. Defensins act mainly on the cell membranes of pathogenic microorganisms, binding to sphingolipids on the outer side of phospholipids. Defensins are able to disrupt the cell membranes of certain fungi, causing changes in the anions and cations on the cell membrane. Thus defensins disrupt the cell membrane of pathogenic microorganisms, altering their permeability and creating phenomena such as Ca^2+^ inward flow, K^+^ outward, and medium liquefaction (Shahmiri et al., 2023).

Most phytodefensins are resistant to fungal pathogens and ward off diseases caused by fungi. It has been found that most defensins are induced to be expressed by fungal infection. GXCX3-9C has antifungal activity and is a core motif in plant defensins. (Sagaram et al., 2011). The core motif peptides of defensins in Medicago truncatula are MtDef4 and MtDef5. And these defensins show high activity against both plant and human bacterial pathogens (Andrew Edward Sathoff et al., 2019). The carrot defensin Rs-AFP2 binds to the nerve sphingolipid glucosylceramide (GlcCer) and acts on the cell membrane, with the binding site being the sphingolipid on the outside of the phospholipid. This in turn disrupts the cell membrane and alters its permeability, resulting in Ca^2+^ in-flow, K^+^ out-flow, and liquefaction of the culture medium (Sadhu et al., 2023). In addition, it has been shown that the tolerance of certain plants to the heavy metal zinc is related to phyto-defensin. Reduced Expression of Plant Defensin 1 in Arabidopsis Leads to Increased Resistance to Pathogenic Bacteria and Zinc Toxicity (Nguyen et al., 2023). Phytodefensins have a very broad development prospect as a new antifungal drug. Research has shown that plant-derived AMPs can be used as alternative molecules to overcome pathogen resistance (Lima et al., 2022). This property can therefore be utilized to research new drugs to fight cancer. *Candida otitis* is a pan-resistant pathogenic yeast that can treat immunocompromised patients. Phytodefensins can influence the virulence properties of clinical strains of *C. auris* (Kamli et al., 2022). Plant defensins PaDef and γ-thionin are potential angiogenicmodulators of the VEGF activity on endothelial cells (Falcón-Ruiz et al., 2023). Therefore, plant defensins, with their broad-spectrum, antimicrobial and high efficiency, provide new ideas for the research and development of new antifungal and antitumor drugs. With the in-depth exploration of its mechanism of action, it is expected to be put more into the industrial, medical and other fields to play a role.

Wheat is one of the major grain crops in China and has an important position in food production, with more than 1/3 of the world’s population taking it as their staple food. Since the growth of wheat is attacked by diseases, it is necessary to tap the relevant genes to breed disease-resistant varieties. A review of the literature shows that the *PDF* gene family is more fully reported in Arabidopsis. *PDF* gene family analysis is not currently reported in wheat, maize and rice. In study, using Arabidopsis PDF proteins (Araport11) as query, *PDF* genes in wheat were identified. Moreover, PDF protein characters and the phylogenetic relationships were analyzed. The function of *PDF* was explored through a series of analyses including chromosomal localization, conserved structure and gene structural domains of the wheat *PDF* gene. This study provides the first systematic certification and classification of the *PDF* genes family in wheat and its subgenomic donors. Genome-wide identification of *TaPDFs* will enrich the study of plant disease resistance genes and provide a basis for wheat varieties breeding.

## Materials and methods

### Screening and analysis of *PDF* genes in wheat, maize, and rice genomes

The 15 protein sequences from Arabidopsis *PDF* family were collected from TAIR database (http://www.Arabidopsis.org/index.jsp) (Mäser et al., 2001), and served as query sequences to research *PDF* genes in wheat genome (IWGSC v1.1), Maize genome (maizeGDB, B73 RefGen_v5, https://www.maizegdb.org/), and rice genome (RGAP, RGAP7, http://rice.plantbiology.msu.edu/index.shtml) using BLASTp with e-value < 10^−10^ (Clavijo et al., 2017). Subsequently, the PDF proteins containing the major intrinsic protein structural domain (gamma – thiionin, PF00304) were retained after searching in the Pfam database (http://pfam.xfam.org/) (Finn et al., 2016).

The length of CDS region, molecular weight (MW), isoelectric point (pI), stability, and hydrophilicity characteristics (GRAVY) of PDF proteins in wheat, maize, and rice were analyzed using ExPASy Server 10 (https://prosite.expasy.org/PS50011) (Li et al., 2018). Signal peptide of wheat *PDFs* was predicted by SignalP 4.1 (http://www.cbs.dtu.dk/services/SignalP/). The subcellular localization of wheat PDFs was predicted by WoLF PSORT (https://wolfpsort.hgc.jp/) and Plant - mPLoc (http://www.csbio.sjtu.edu.cn/bioinf/plant-multi/).

### Phylogeny, chromosome localization, conserved motif, gene structure, and *cis*-acting elements analysis

Comparison of all protein sequences from wheat, rice, maize and Arabidopsis using ClustalW2 (Thompson et al., 1994). Methods Phylogenetic trees were constructed with MEGA 7.0 and calculated according to the maximum likelihood (ML) method (Kumar et al., 2016). Drawing and landscaping the phylogenetic tree was accomplished through the Interactive Tree of Life website (IToL, version 3.2.317, http://itol.embl.de). *TaPDF* gene annotation file containing information on the chromosome position were extracted from the genome annotation information GFF3 file. Afterwards the physical map was then plotted using MapInspect software (Hu and Liu, 2011). *TaPDF* genome annotation information was used to map the gene (exon-intron) structure using GSDS2.0 (http://gsds.cbi.pku.edu.cn/index.php) (D.-M. Huang et al., 2022). The conserved motifs of TaPDF proteins were identified by MEME (v4.9.1, http://meme-suite.org/index.html) (Zheng et al., 2017). The conserved motifs are identified by the following criteria: (1) All sequences can include multiple non overlapping motifs; (2) Up to 20 different motifs; (3) The length of the motif is 6-50 aa. The protein sequences were uploaded to SWISS-MODEL website (https://www.swissmodel.expasy.org/) in order to identify protein structure with default parameters (Arnold et al., 2005).

The 1500 bp sequence upstream of the start codon of *TaPDFs* were submitted to the PlantCARE database to predict the *cis*-acting elements (Lescot et al., 2002). The enrichment analysis of *cis*-acting elements was performed using the pattern enrichment analysis (AME) function in the MEME program to identify the regulatory elements (İlhan et al., 2018).

### Homology analysis

Genomic data for *T. dicoccoides* (v1.0.43), *Ae tauschii* (v4.0.43), and *T. urartu* (v1.43) was obtained from the EnsemblPlants database (http://plants.ensembl.org/index.html) (Alaux et al., 2018). The *PDF* homologous of urartu wheat (*T. urartu*), wild dicoccoides (*T. dicoccoides*), and rough goat grass (*Ae. tauschii*) were identified by BLASTp (threshold E<10^-10^, match >80%). The protein which contains the structural domain of PF00304 (Gamma-thionin) was identified as *PDF* gene family member. The sequences of the identified *PDFs* were determined by whether the fragments were duplicated or tandem duplicated on the same chromosome, and homology mapping was performed using the R package “circlize”.

### Transcriptome analysis of *TaPDFs* gene and qRT-PCR analysis

In order to further study the expression patterns of *TaPDF* genes under different stresses, original RNA-seq data under various conditions was downloaded from the SRA database from NCBI (**Appendix S1**), and was mapped to the wheat reference genome using hisat2. The gene expression level was calculated by Cufflinks, which was normalized through the fragment by the exon base number per kilogram (FPKM) model per million bases (Trapnell et al., 2012). Finally, pheatmap package was used to generate the heatmap of *TaPDF* genes.

RNA was extracted from wheat leaves using TRIzol™ Reagent (Invitrogen). The cDNA obtained by reverse transcription was used as a template to amplify the target genes. The six highly expressed *TaPDF* genes *TaPDF4.9*, *TaPDF5.4*, *TaPDF2.12*, *TaPDF2.15*, *TaPDF2.23*, and *TaPDF2.20*, which were highly expressed in the transcriptome analysis, were selected for qRT-PCR analysis during *Fusarium graminearum* infestation. The wheat varieties used in this experiment were Yangmai 158 and Xinong 98710. The primers used in this study were listed in **Appendix S2**. The screened genes were analyzed by qRT-PCR on a CFX 96 Real-Time PCR System (Bio-Rad). (Mamidala et al., 2011). The fluorescence quantification reaction system consisted of 10 μL of 2× SYBR Green (Vazyme), 0.4 μL of each of the upstream and downstream primers, 2 μL of 50 ng/μL cDNA, and the addition of 7.2 μL of ddH_2_O, for a total volume of 20 μL. The reaction conditions were 95°C for 3 min, 95°C for 5 s, primer annealing/extension, and 58°C for 30 s. A total of 45 cycles were started from step 2. Each gene requires the use of three technical repeats. The final resultant data were used to determine relative expression levels using the 2-ΔΔCt method (Livak and Schmittgen, 2001). Use of the ADP-ribosylation factor Ta2291, stably expressed under various conditions, as an internal reference gene for qRT-PCR analysis (Huang et al., 2021).

### Inoculation of pathogenic phytophthora and subcellular localization

The target gene were ligated into the pART27 and pART27 vector with YFP using a homologous recombination kit (Vazyme). The connected *TaPDF2.15* and *TaPDF4.9* were selected for transient expression in *Nicotiana benthamiana* leaves. After two to three days, the leaves were cut off, and inoculated with *Phytophthora infestans* strain ‘88069’, then they were placed in a humidor infecting box, stored with thetemperature at 20 °C. After four to six days, measure the lesion diameter of the inoculated leaves were measured (measure the longest and shortest). GFP signals from *TaPDF4.9*, *TaPDF5.4*, *TaPDF2.12*, and *TaPDF2.15* trans-overexpressed leaves were observed two days after injection using laser scanning confocal microscope (Leica TCS SP8).

## Result

### Screening and identification of *PDF* genes in wheat, maize, and rice genomes

**Figure 1:**
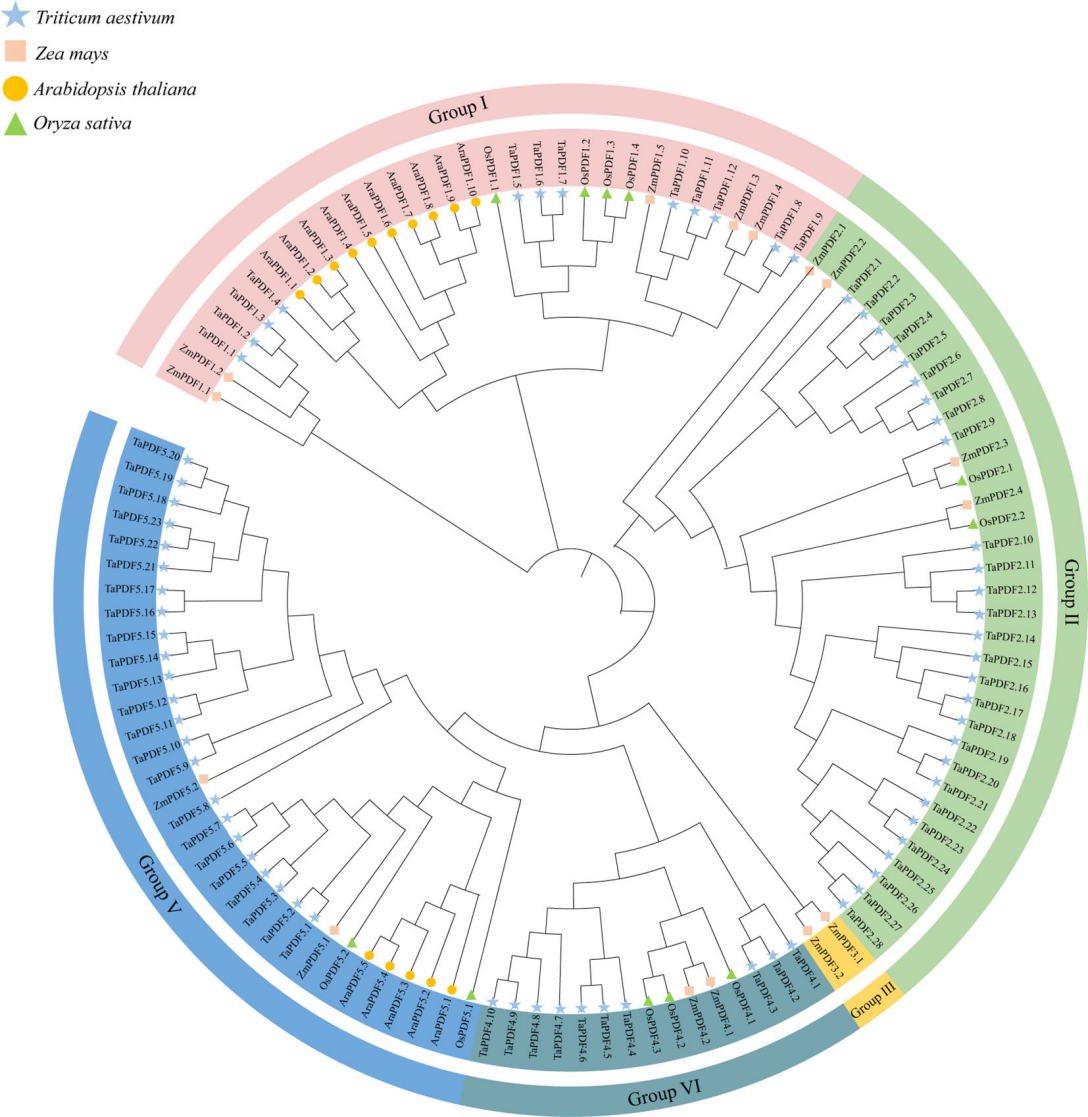
Phylogenetic tree of the PDF family of Triticum aestivum, Zea mays, Oryza sativa, and Arabidopsis thaliana.

The sequences containing the conserved domain of PF00304 (Gamma-thionin) were retained using Pfam online tool. Finally, 73 wheat *PDF* members, 15 members of maize, and 11 members of rice were identified (**Table 1**). The *PDF* genes of the four species were divided into five groups according to the rootless phylogenetic trees (**Figure 1**). The genes are named according to the location of the components, e.g. the first gene located in Group I is named as *TaPDF1.1*. Most of these *TaPDFs* are concentrated in Group II, IV and V. Group III is only *ZmPDF*.

**Table 1:** Protein characteristics of wheat PDF family.

					Instability index			Predicted location(s)
Name	Accession numbers	Length/aa	Molecular	Isoelectric point		Aliphatic index	GRAVY	
			mass/kDa					
TaPDF1.1	TraesCS6A02G157100.1	86	9.55	5.55	64.93	70.35	-0.134	Vacuole
TaPDF1.2	TraesCS6B02G184900.1	86	9.49	5.55	61.52	73.72	-0.117	Vacuole
TaPDF1.3	TraesCS6D02G146400.1	86	9.55	5.48	56.35	69.19	-0.205	Vacuole
TaPDF1.4	TraesCS6A02G126100.1	78	8.71	8.09	45.8	97.44	0.41	Nucleus
TaPDF2.1	TraesCS5B02G329100.1	77	8.4	9.41	55.84	78.57	-0.044	Vacuole
TaPDF2.2	TraesCS5A02G329000.1	77	8.28	9.08	33.58	83.64	0.058	Vacuole
TaPDF2.3	TraesCS5D02G334900.1	77	8.38	9.24	39.41	92.47	0.053	Vacuole
TaPDF2.4	TraesCS3D02G444200.1	128	14.33	8.7	64.43	63.91	-0.58	Nucleus. Vacuole.
TaPDF2.5	TraesCS3B02G488800.1	111	12.2	8.86	60.16	49.28	-0.45	Vacuole
TaPDF2.6	TraesCS5A02G329100.1	81	8.73	8.95	37.95	68.64	0.001	Nucleus
TaPDF2.7	TraesCS5B02G329200.1	80	8.77	8.8	42.58	64.62	-0.054	Nucleus. Vacuole.
TaPDF2.8	TraesCS5D02G335000.1	80	8.62	8.62	35.79	72	0.089	Nucleus
TaPDF3.1	TraesCS2B02G062000.1	75	7.85	5.72	70.96	79.33	0.299	Vacuole
TaPDF3.2	TraesCS2D02G048200.1	75	8.03	5.75	69.96	79.33	0.249	Vacuole
TaPDF3.3	TraesCS2D02G047900.1	75	8.04	5.75	69.83	78	0.22	Vacuole
TaPDF3.4	TraesCS2A02G049000.1	75	7.95	5.28	72.4	78	0.269	Vacuole
TaPDF3.5	TraesCS2A02G048900.1	78	8.39	8.48	47.07	85	0.385	Vacuole
TaPDF3.6	TraesCS2D02G047800.1	78	8.37	8.48	45.41	86.28	0.39	Vacuole
TaPDF3.7	TraesCS2B02G062100.1	78	8.36	8.48	42.82	85	0.373	Vacuole
TaPDF3.8	TraesCS5A02G525600.1	71	7.8	5.59	43.78	68.73	0.351	Nucleus. Vacuole.
TaPDF3.9	TraesCS4D02G349900.1	76	8.17	7.52	45.98	83.42	0.521	Vacuole
TaPDF3.10	TraesCS4B02G356200.1	76	8.09	8.17	46.61	88.55	0.621	Vacuole
TaPDF3.11	TraesCS3B02G476300.1	74	8.12	8.13	33.15	78.95	0.193	Vacuole
TaPDF3.12	TraesCS3A02G442500.1	74	8.13	8.46	38.15	84.19	0.264	Vacuole
TaPDF3.13	TraesCS1D02G388400.1	120	13.07	6.69	44.68	85.92	0.175	Vacuole
TaPDF3.14	TraesCS3D02G435100.1	76	8.44	8.14	21.79	84.47	0.212	Vacuole
TaPDF3.15	TraesCS3A02G442400.1	76	8.42	8.14	20.38	84.47	0.242	Vacuole
TaPDF3.16	TraesCS3B02G476200.1	72	8	8.47	22.45	84.36	0.215	Vacuole
TaPDF3.17	TraesCS3A02G442700.1	69	7.36	6.68	26.89	83.33	0.359	Vacuole
TaPDF3.18	TraesCS3D02G435300.1	75	8.02	6.37	29.13	81.87	0.167	Vacuole
TaPDF3.19	TraesCS7A02G183500.1	70	7.56	8.72	42.16	84.86	0.467	Vacuole
TaPDF3.20	TraesCS7D02G185100.1	70	7.71	7.54	40.13	75	0.283	Vacuole
TaPDF3.21	TraesCS7B02G088300.1	76	8.26	8.72	44.81	81.97	0.295	Vacuole
TaPDF3.22	TraesCS7D02G185000.1	76	8.26	8.72	44.81	81.97	0.295	Vacuole
TaPDF3.23	TraesCS7A02G183400.1	76	8.31	8.72	45.93	79.34	0.301	Vacuole
TaPDF3.24	TraesCS7A02G183600.1	75	8.28	8.71	40.03	79.07	0.309	Vacuole
TaPDF3.25	TraesCS7D02G185200.1	75	8.19	8.49	36.71	76.53	0.337	Vacuole
TaPDF3.26	TraesCS7B02G088400.1	71	7.81	8.16	43.73	80.85	0.37	Vacuole
TaPDF4.1	TraesCS4D02G321100.1	81	8.59	9.33	37.46	79.63	0.127	Vacuole
TaPDF4.2	TraesCS5A02G496500.1	78	8.35	9.37	45.59	87.41	0.132	Vacuole
TaPDF4.3	TraesCS4D02G321000.1	82	9.09	9.68	62.49	70.2	-0.157	Vacuole
TaPDF4.4	TraesCS4B02G324000.1	82	9.08	9.6	63.91	70.24	-0.16	Vacuole
TaPDF4.5	TraesCS5A02G496600.1	82	8.95	9.69	26.52	67.93	-0.133	Vacuole
TaPDF4.6	TraesCS4B02G324100.1	82	8.91	9.49	26.87	66.71	-0.144	Vacuole
TaPDF4.7	TraesCS4D02G321200.1	82	8.93	9.69	27.84	66.71	-0.151	Vacuole
TaPDF4.8	TraesCS2B02G396100.1	85	9.86	8.91	33.9	55.06	-0.455	Vacuole
TaPDF4.9	TraesCS1A02G013600.1	82	8.97	8.9	46.25	73.9	-0.017	Nucleus. Vacuole.
TaPDF4.10	TraesCS1D02G012600.1	82	8.91	8.72	47.81	73.78	-0.072	Vacuole
TaPDF4.11	TraesCS1A02G014800.1	82	8.95	9.92	72.68	72.68	-0.084	Vacuole
TaPDF4.12	TraesCS1B02G017700.1	77	8.38	8.72	40.66	76.1	0.035	Vacuole
TaPDF4.13	TraesCS1D02G012100.1	76	8.42	7.56	31.42	64.21	-0.213	Vacuole
TaPDF4.14	TraesCS1B02G018100.1	81	8.98	8.5	58.07	59.26	-0.265	Vacuole
TaPDF4.15	TraesCS7B02G244000.1	83	9.17	8.16	37.04	60.12	-0.212	Vacuole
TaPDF4.16	TraesCS1D02G012000.1	82	9.06	8.7	43.39	28.41	-0.198	Vacuole
TaPDF4.17	TraesCS1A02G014100.1	74	8.42	6.79	23.04	27.7	-0.677	Vacuole
TaPDF4.18	TraesCS1A02G014000.1	82	9.1	7.6	44.51	64.27	-0.204	Vacuole
TaPDF4.19	TraesCS1D02G011900.1	86	9.41	8.17	45.61	66.05	-0.221	Vacuole
TaPDF4.20	TraesCS1B02G018000.2	79	8.69	7.57	49.64	54.43	-0.201	Vacuole
TaPDF4.21	TraesCS1B02G018000.1	82	9.14	8.48	59.81	53.66	-0.265	Vacuole
TaPDF4.22	TraesCS6B02G103000.1	82	8.89	9.08	58.38	70.37	0.113	Vacuole
TaPDF4.23	TraesCS6B02G102900.1	82	8.82	9.22	40.65	81.1	0.155	Vacuole
TaPDF4.24	TraesCSU02G027100.1	82	8.83	9.22	39.15	82.2	0.189	Vacuole
TaPDF4.25	TraesCSU02G026600.1	82	8.83	9.22	39.15	82.2	0.189	Vacuole
TaPDF4.26	TraesCS6A02G076800.1	82	8.76	9.22	43	79.88	0.195	Vacuole
TaPDF4.27	TraesCS1A02G050700.1	82	8.82	8.5	66.51	72.68	0.054	Vacuole
TaPDF4.28	TraesCS1D02G053000.1	82	8.94	9.1	64.46	63.29	-0.14	Vacuole
TaPDF4.29	TraesCS1B02G067300.1	82	8.86	9.1	70.47	71.59	-0.015	Vacuole
TaPDF4.30	TraesCS1A02G050900.1	82	8.87	8.92	63.07	77.56	0.148	Vacuole
TaPDF4.31	TraesCS1A02G050800.1	82	8.98	9.08	54.27	66.83	-0.079	Vacuole
TaPDF4.32	TraesCS1D02G052900.1	81	8.89	8.72	63.95	65.19	-0.07	Vacuole
TaPDF4.33	TraesCS1B02G067100.1	84	9.29	8.43	63.35	70.95	-0.01	Vacuole
TaPDF4.34	TraesCS1B02G067000.1	82	8.92	8.49	59.01	79.88	0.143	Vacuole
TaPDF4.35	TraesCS1A02G050600.1	82	9.02	9.08	58.82	73.9	-0.01	Vacuole
TaPDF4.36	TraesCS1D02G052800.1	82	8.97	8.9	49.74	72.68	-0.024	Vacuole

As shown in **Table 1**, the average length of *TaPDF* is 80.57 aa. The average molecular weight of TaPDFs is 8.8 KD, and the average isoelectric point is 8.27, which indicates that TaPDFs are alkaline proteins. The average instability index is 47.12, which reveals the TaPDFs are unstable proteins. The average value of the aliphatic index is 73.67. The average value of hydrophilicity is 0.06. Multi website prediction of subcellular localization results shows that most TaPDF proteins are located in vacuoles, with a small portion located in the nucleus.

### Chromosomal mapping and gene duplication analysis of *TaPDF* genes

**Figure 2:**
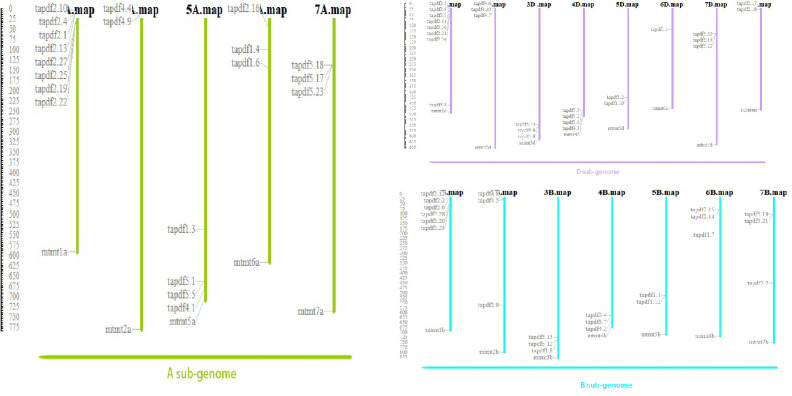
Chromosomal location of wheat *PDF* family.

According to the chromosome distribution map (**Figure 2**), *PDF* genes were found to be widely distributed on each chromosome, with each chromosome containing multiple genes. As shown in **Figure 2**, wheat PDF contains multiple genes on its chromosomes during evolution. And each chromosome has duplicate genes. This shows that segmental duplication is the main reason for the expansion of the PDF family during the evolution of wheat.

### Conserved motif and gene structure analysis of *TaPDF* gene family

**Figure 3:**
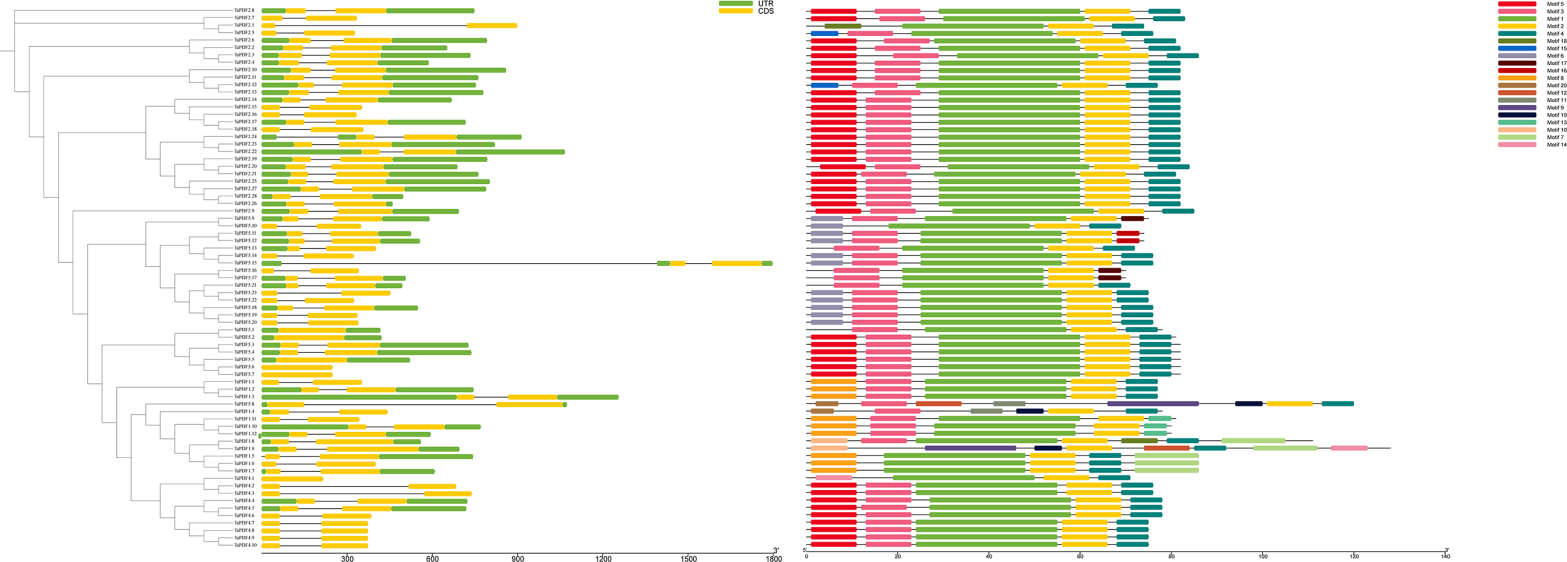
The evolutionary tree, motif analysis and gene structure analysis of 74 wheat *PDF* families.

Based on the *TaPDF* genomic information, the gene structure was mapped using GSDS2.0 (**Figure 3**). The vast majority of *TaPDF* genes contain non-coding regions at the 5’ and 3’ ends, and only a few *TaPDFs* do not contain non-coding regions. Most *TaPDF* genes contain two extrons.

TaPDFs all contain motifs 1, 2, 14 and 18. The sequence, position and identification of the conserved motifs in the TaPDF protein are shown in **Figure 3**. All identified wheat genes contain conserved structural domains of the PDF family. The wheat *PDF* gene family contains characterized amino acids, including a series of highly conserved active site residues consistent with previous studies on different plant species.

### Identification of *PDF* members in common wheat, urartu wheat, wild emmer wheat, and coarse goat grass

**Figure 4:**
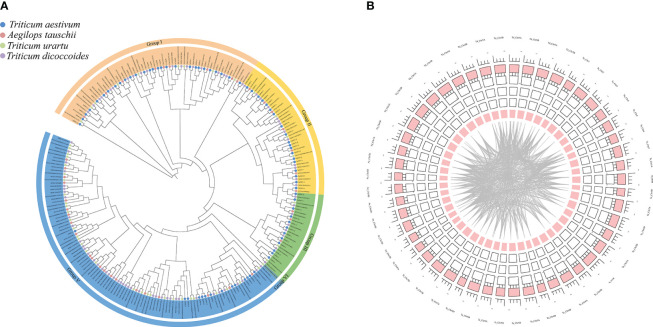
Phylogenetic tree and joint analysis circle diagram of the PDF family in T. aestivum, T. urartu, T. dicoccoides and Ae. tauschii. **(A)** is the T. aestivum, T. urartu, T. dicoccoides and Ae. tauschii evolutionary tree. **(B)** is the wheat homology analysis.

A phylogenetic tree was constructed using MEGA 7.0 maximum likelihood (ML) method (**Figure 4A**). PDF proteins can be divided into five groups, of which groups I and V contain more PDF protein members. By gene duplication events, 2940 duplicate pairs were obtained. The common wheat positional information was put into the R package for circles plot analysis (**Figure 4B**). The results suggest that *PDFs* from each genomic donor may be ancestrally similar to each other. Alternatively, initially different *PDFs* may be stabilized after a long domestication process and lead to alterations in protein structure and function. Homology analysis of *TaPDF* suggests that wheat may be repeated multiple times in polyploidy, and we hypothesize that this may have made wheat more adapted to its environment and increased the number of wheat *PDF* genes during evolution.

### *Cis*-acting elements of *TaPDFs* gene

**Figure 5:**
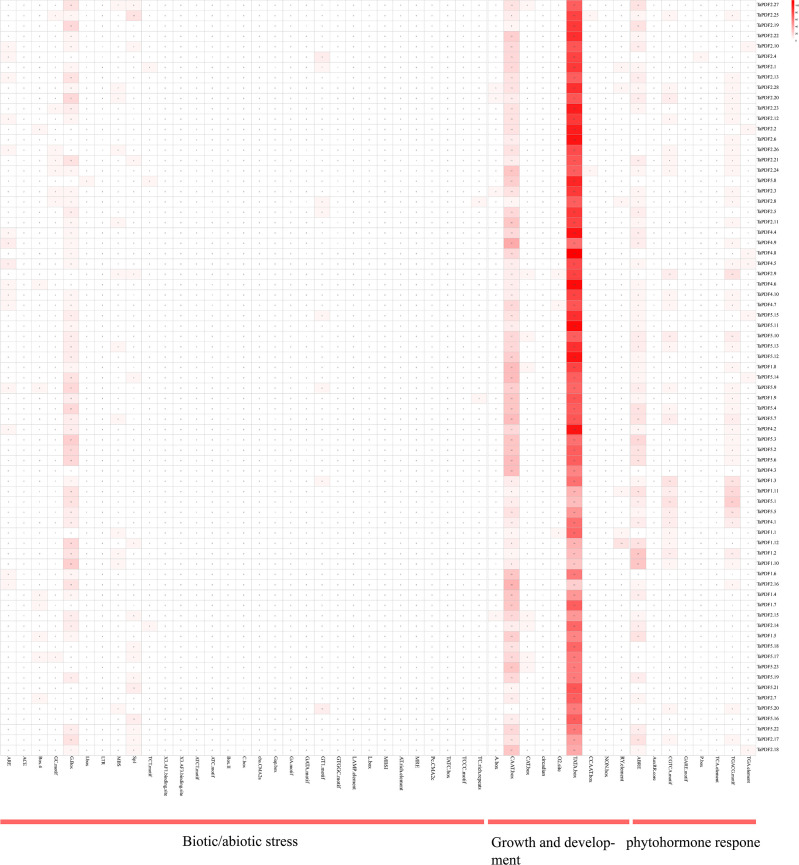
Cis-acting elements in wheat *PDF* gene promoter.

A total of 60 *cis*-acting elements were screened by intercepting a 1500 bp region upstream of the *TaPDF* gene. These components are divided into three categories: phytohormones, biotic stress-abiotic stress, and growth and development (**Figure 5**). The results indicate that the promoter sequence of *TaPDF* gene has multiple cis-regulatory elements, including *cis*-acting elements involved in defense and stress response, light-responsive elements, low-temperature-inducible elements, anaerobic-inducible elements, and drought-inducible elements biotic-abiotic taxa. The most abundant original in *TaPDF* is CAAT-box as promoter-associated original. The most widely distributed *cis*-element is the abscisic acid-responsive *cis*-acting element ABRE. The two core promoter elements of the same growth and development-related element are TATA-box and CAAT-box. CGTCA motifs and TGACG motifs are the major motifs of jasmonate methyl ester response elements. The most common *cis*-element among all genes was the jasmonate methyl ester response element. And the light-responsive elements (g-boxes) are also more widely distributed. The next most common *cis*-elements are the methyl jasmonate response elements (CGTCA motif and TGACG motif), which are distributed in almost all genes. It suggests that *PDF* is associated with plant growth and development in wheat, and may also be associated with plant disease resistance.

### Multi-conditional transcriptome of *TaPDF* genes

**Figure 6:**
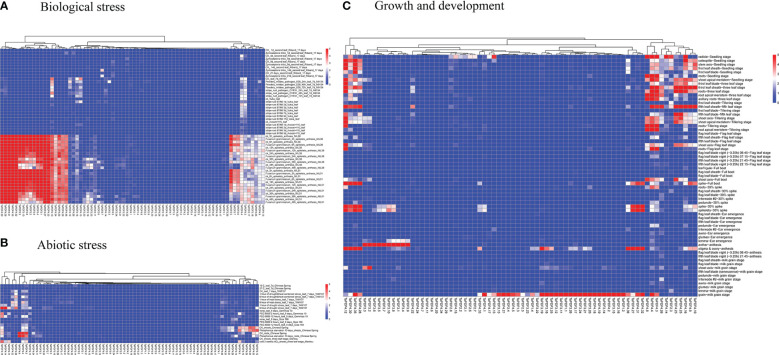
Heat map of growth and development of wheat *PDF* gene family. **A**, **B** and **C** represents transcriptomic data of TaPDFs in terms of biotic stress, abiotic stress and growth and development.

The transcriptome data downloaded and collated from the NCBI website were divided into three categories, including growth and development, abiotic stresses, and biotic stresses. As shown in the heat map in **Figure 6**, the overall expression of wheat *PDF* genes was low in the early stages of growth and development. *TaPDF5.4* was highly expressed at the apical meristem tiller stage, apical meristem trilobal stage, radicle seedling stage, root seedling stage, root tiller stage, root leaf stage, root trilobal stage and shoot apex meristem seedling stage. Therefore, the function of *TaPDF5.4* may be inseparably related to root growth and development. The overall expression of wheat *PDF* genes was also lower during abiotic stress. However, during the biotic stress treatment, *TaPDF4.9*, *TaPDF5.4*, *TaPDF2.12*, *TaPDF2.15*, *TaPDF2.23*, and *TaPDF2.20* were more highly expressed in spikelets inoculated by *Fusarium graminearum* at 3, 6, 12, and 24 h. The results suggest that the *TaPDF* genes may be associated with plant disease resistance, which was consistent with the prediction of *PDF* genes function.

### Expression analyses of *TaPDF* genes

**Figure 7:**
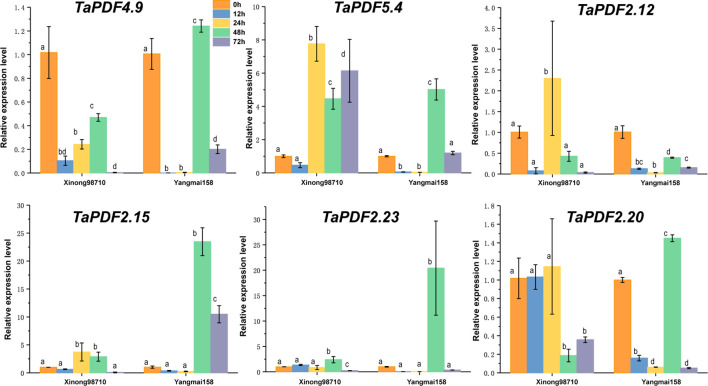
Expression patterns of six wheat (Triticum aestivum) *TaPDF* genes under *F. graminearum* treatments.

To further understand the role of *TaPDF* genes in reaction to biotic stresses, six highly expressed genes (*TaPDF2.12*, *TaPDF2.15*, *TaPDF2.20*, *TaPDF2.23*, *TaPDF4.9*, and *TaPDF5.4*) in the transcriptome (**Figure 6**) were selected. Expression of PDF genes in Yangmai 158 (disease-resistant) and Xinong 98710 (disease-susceptible) after inoculation with *F. graminearum* was analyzed by qRT-PCR (**Figure 7**). The overall expression level of *TaPDF* genes were up-regulated after inoculation of *F. graminearum* on the coleoptiles of two wheat species. However, *TaPDF4.9* gene expression level was down-regulated in Xinong 98710 and *TaPDF2.21* gene expression level was in a down-regulated state in Yangmai 158. According to the quantitative results, *PDF* gene may play different roles in wheat at 24h and 48h of *F. graminearum* infestation.

### Subcellular localization of *TaPDF* genes

**Figure 8:**
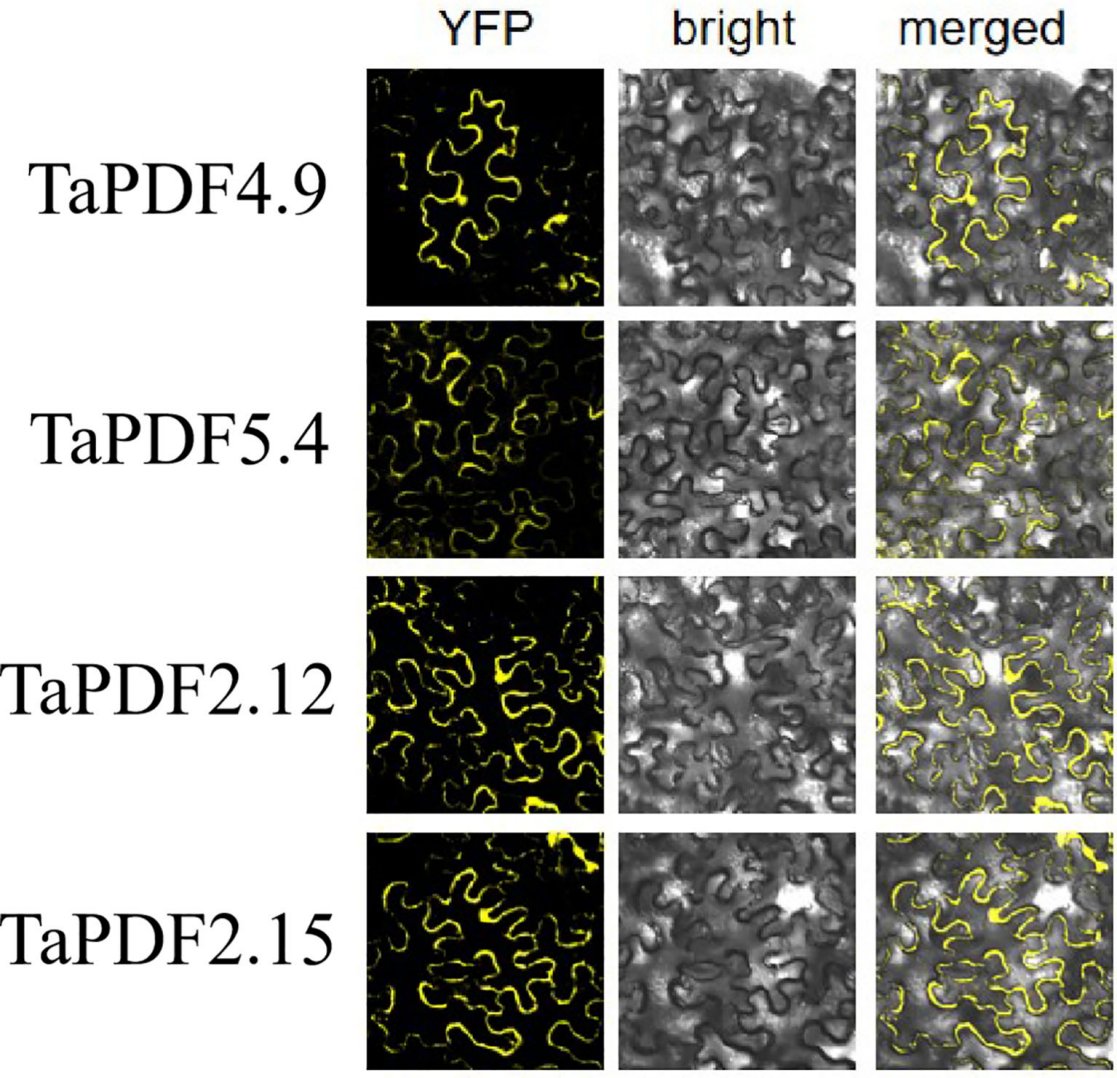
Subcellular localization of TaPDF4.9, TaPDF5.4, TaPDF2.12 and TaPDF2.15.

Most *PDF* genes were predicted to be located in vesicles, whereas only four genes were predicted to be on vesicles and nucleus. *TaPDF4.9*, *TaPDF5.4*, *TaPDF2.12*, and *TaPDF2.15* genes were selected as target genes for homologous recombination with the digested vector pART27. The results of **Figure 8** showed that the four target genes were basically localized to the cytoplasm and cell membrane, and *TaPDF4.9* was also localized in the nucleus.

### Agrobacterium-mediated transient overexpression and inoculation of *Phytophthora infestans*

**Figure 9:**
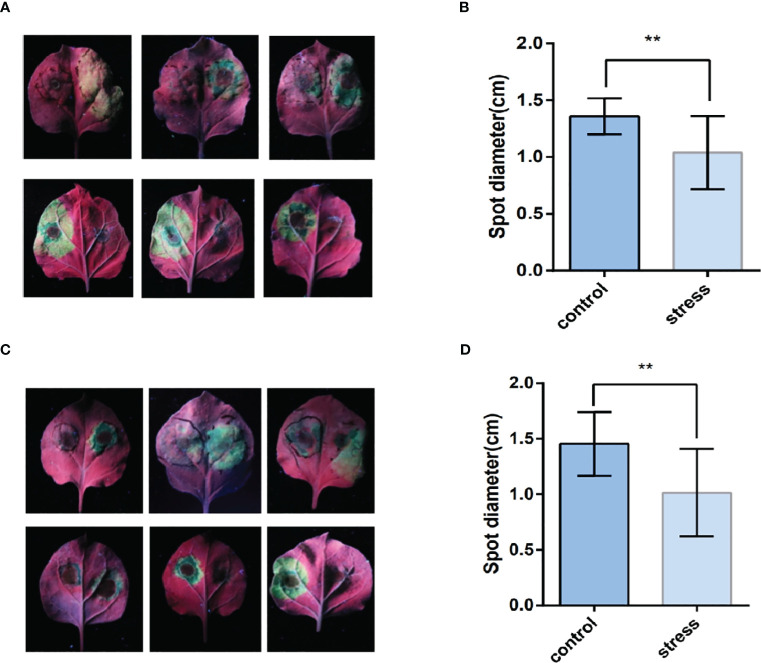
The leaf surface mediated by *TaPDF4.9* (AB) and *TaPDF2.15* (CD) inoculation with Phytophthora infestans. **(A)** is TaPDF4.9 infestation on tobacco, **(B)** is TaPDF4.9 infestation length. **(C)** is TaPDF2.15 infestation on tobacco and **(D)** is TaPDF2.15 infestation length. ** represents highly significant differences.

After four to six days of inoculation with *Phytophthora infestans*, the lesion diameter was measured (**Figures 9A, C**). As shown in **Figures 9B, D**, the lesion area on the leaves, where *TaPDF2.15* and *TaPDF4.9* were transient overexpressed, was smaller (P value < 0.01) than the control. This result suggests that *TaPDF* may play positive roles in resistance to *Phytophthora infestans* strain ‘88069’.

## Discussion

Plant defensins are widely distributed in the leaves, fruits, roots, stems, seeds, and tubers. Based on the genome-wide characterization of *PDFs* already performed in the literature in Arabidopsis thaliana (Mäser et al., 2001). However, there is a lack of systematic studies on the *PDF* family in wheat. In order to gain a deeper understanding of the functioning of wheat *PDFs*, this paper examines them. For the identification of family members, BLASTp searches were performed using known Arabidopsis PDF protein sequences (Kushmerick et al., 1998) and conserved structural domain sequences as query sequences (Clavijo et al., 2017). Sequences were queried in the wheat genome (IWGSC v1.1) and candidate hits were further checked by Pfam and InterProScan to exclude sequences without the Gamma - thiionin structural domain. Seventy-three wheat PDF proteins were finally identified. This study also identified *PDF* genes in rice and maize, which contained 15 maize *PDF* genes and 11 rice *PDF*s. The number of *PDF* genes varies in different plants. Differences in the number of genes from other species indicate functional redundancy in *TaPDFs*. However, genes do not correspond to changes in genome size.

Higher numbers of *PDFs* were identified in wheat than in Arabidopsis, maize and rice. The results illustrate the complexity of genetic traits in wheat. Homology analyses of *TaPDF* indicate that wheat may repeat multiple times in polyploidy, which we hypothesize may have made wheat more environmentally adapted and increased the number of wheat *PDF* genes during evolution. Based on gene structure and protein physicochemical property analyses, the wheat *PDF* gene has a highly conserved gene structure containing eight conserved cysteine residues forming four pairs of disulfide bonds (Cys4-Cys7, Cys3-Cys6, Cys2-Cys5, and Cys1-Cys8) (Thomma et al., 2002). The conclusions in the article are the same as those previously reported for the *PDF* family having the same structure. And the molecular weight size just shows that wheat *PDF* belongs to the shorter peptides with smaller molecular weight. The abscisic acid-responsive cis-acting element ABRE, the core promoter elements TATA box and CAAT box in the promoter of the wheat *PDF* gene are all growth and development-related elements. This suggests that *TaPDF* is associated with plant growth and development. Whereas methyl jasmonate response elements are distributed in almost all genes, we can infer that *TaPDF* is associated with plant disease resistance. It has been shown that plant defensins are commonly considered to display antimicrobial activity against only fungi (Sathoff et al., 2019). To further reveal the function of *TaPDF*, six genes *TaPDF2.12*, *TaPDF2.15*, *TaPDF2.20*, *TaPDF2.23*, *TaPDF4.9* and *TaPDF5.4* were analyzed by qRT-PCR. The expression levels of *TaPDFs* were differentially up-regulated by *Fusarium graminearum*. The above results indicate that *TaPDFs* are not only involved in wheat growth and development, but also play a positive regulatory role in the stress response process. The effect of *TaPDF* on disease resistance was analyzed through the resistance of different wheat varieties to *F. graminearu*. The results showed that *TaPDF* has an effect on plant disease resistance. Relevant studies have shown that PsDef1 is significantly increased in Scots pine seedlings during germination and in their response to pathogenic infection with Heterobasidion annosum (Kovaleva et al., 2011).

And after inoculation with Phytophthora infestans, it can be seen that the plaque becomes smaller, indicating that both selected *TaPDF2.15* and *TaPDF4.9* can inhibit Phytophthora infestans. It shows that AhPDF1s and AtPDF1s were able to confer Zn tolerance and AhPDF1s also displayed antifungal activity (Shahzad et al., 2013). It can be seen that the *TaPDF* should be functionally similar to that in Arabidopsis thaliana, both of which have the function of inhibiting the infestation of pathogenic bacteria. Although the mechanism of amplification of such gene families is well understood in this paper, the genes are not well understood in different species, which will have to be studied in further depth. The gene family aspect could be further systematically analyzed for defensin gene families by combining more species. The results of subcellular localization showed that the selected genes were basically localized to cytoplasm and cell membrane, but the multisite predictions of the proteins were all on the vesicles and a few on the nucleus. The results indicate that by Agrobacterium alone mediated *Nicotiana benthamiana* leaves could not be accurately localized to the organelles, but also require organelle-corresponding stains, which need to be investigated in subsequent work.

## Conclusions

In this study, we identified 73 wheat *PDF* family members using bioinformatics methods, 15 members of corn *PDF* genes and 11 members of rice *PDF* genes. With the hexaploid wheat as the main research object and the amino acid sequence of *PDF* identified in Arabidopsis (Kushmerick et al., 1998), maize, and rice as reference, 35, 65, and 34 *PDF* gene family members were identified in the genomes of *T. dicoccoides*, *Ae. tauschii*, and *T. urartu*, respectively. As can be seen from the basic physicochemical properties, most of the TaPDFs are basic proteins and most are unstable. The genes are relatively evenly distributed across almost every chromosome. The high number of gene duplicate pairs indicates possible redundancy of function. The subcellular localization results of *TaPDF4.9*, *TaPDF5.4*, *TaPDF2.12*, and *TaPDF2.15* are basically located in the cytoplasm and cell membrane, and *TaPDF* 4.9 is also located on the nucleus. Transient overexpression of *TaPDF4.9* and *TaPDF2.15* could inhibit the infection of Phytophthora infestans strain ‘88069’, which has the function of inhibiting the infection of Phytophthora infestans. According to the expression pattern, *TaPDF* is resistant to *F. graminearum*, and most of the selected genes showed an increase in transcript levels after being infested with *F. graminearum*. In this experiment we performed bioinformatic analysis of the wheat PDF family with subcellular localization, quantitative analysis and pathogenicity analysis. Wheat PDF was found to be effective in suppressing late blight in the Phytophthora infestans. As the genomes of more and more species are sequenced, it is believed that more members of the plant defensin family will be identified. With the development of functional genomics and proteomics, the role of such genes in wheat will become clearer and their mechanism of action will become clearer. Meanwhile, plant defensins have a very vast development prospect as a new antifungal drug, and researchers have also noticed the potential of plant defensins in the development of new anticancer drugs. Therefore, plant defensins, with their broad-spectrum, antimicrobial and high efficiency characteristics, provide new ideas for the research and development of new antifungal and antitumor drugs.

## Data availability statement

The datasets presented in this study can be found in online repositories. The names of the repository/repositories and accession number(s) can be found in the article/**Supplementary Material**.

## Author contributions

YD: Writing – original draft, Writing – review & editing. YW: Writing – original draft, Writing – review & editing. MT: Writing – original draft, Writing – review & editing. WC: Data curation, Investigation, Validation, Writing – review & editing. YC: Formal Analysis, Methodology, Writing – original draft. WW: Formal Analysis, Project administration, Writing – original draft.

## References

[B1] Alaux M. Rogers J. Letellier T. Flores R. Alfama F. Pommier C.. (2018). Linking the International Wheat Genome Sequencing Consortium bread wheat reference genome sequence to wheat genetic and phenomic data. Genome Biol. 19 (1), 111. doi: 10.1186/s13059-018-1491-4 30115101 PMC6097284

[B2] Arnold K. Bordoli L. Kopp J. Schwede T. (2005). The SWISS-MODEL workspace: a web-based environment for protein structure homology modelling. Bioinformatics 22 (2), 195–201. doi: 10.1093/bioinformatics/bti770 16301204

[B3] Clavijo B. J. Venturini L. Schudoma C. Accinelli G. G. Kaithakottil G. Wright J. Clark M. D.. (2017). An improved assembly and annotation of the allohexaploid wheat genome identifies complete families of agronomic genes and provides genomic evidence for chromosomal translocations. Genome Res. 27 (5), 885–896. doi: 10.1101/gr.217117.116 28420692 PMC5411782

[B4] De Coninck B. Cammue B. P. A. Thevissen K. (2013). Modes of antifungal action and in planta functions of plant defensins and defensin-like peptides. Fungal Biol. Rev. 26 (4), 109–120. doi: 10.1016/j.fbr.2012.10.002

[B5] Falcón-Ruiz E. A. López-Meza J. E. Ochoa-Zarzosa A. (2023). The plant defensins PaDef and γ-thionin inhibit the endothelial cell response to VEGF. Peptides 165, 171008. doi: 10.1016/j.peptides.2023.171008 37054894

[B6] Finn R. D. Coggill P. Eberhardt R. Y. Eddy S. R. Mistry J. Mitchell A. L.. (2016). The Pfam protein families database: towards a more sustainable future. Nucleic Acids Res. 44 (D1), D279–D285. doi: 10.1093/nar/gkv1344 26673716 PMC4702930

[B7] Hu L. Liu S. (2011). Genome-wide identification and phylogenetic analysis of the ERF gene family in cucumbers. Genet. Mol. Biol. 34 (4), 624–633. doi: 10.1590/s1415-47572011005000054 22215967 PMC3229118

[B8] Huang D.-M. Chen Y. Liu X. Ni D.-A. Bai L. Qin Q.-P. (2022). Genome-wide identification and expression analysis of the SWEET gene family in daylily (Hemerocallis fulva) and functional analysis of HfSWEET17 in response to cold stress. BMC Plant Biol. 22 (1), 211. doi: 10.1186/s12870-022-03609-6 35468723 PMC9036726

[B9] Huang W. He Y. Yang L. Lu C. Zhu Y. Sun C.. (2021). Genome-wide analysis of growth-regulating factors (GRFs) in Triticum aestivum. PeerJ 9, e10701. doi: 10.7717/peerj.10701 33552727 PMC7821759

[B10] İlhan E. Büyük İ. İnal B. (2018). Transcriptome - Scale characterization of salt responsive bean TCP transcription factors. Gene 642, 64–73. doi: 10.1016/j.gene.2017.11.021 29129811

[B11] Kamli M. R. Sabir J. S. M. Malik M. A. Ahmad A. (2022). Characterization of Defensin-like Protein 1 for Its Anti-Biofilm and Anti-Virulence Properties for the Development of Novel Antifungal Drug against Candida auris&lt;/i&gt. J. fungi (Basel Switzerland) 8 (12), 1298. doi: 10.3390/jof8121298 PMC9786216 36547631

[B12] Kovaleva V. Krynytskyy H. Gout I. Gout R. (2011). Recombinant expression, affinity purification and functional characterization of Scots pine defensin 1. Appl. Microbiol. Biotechnol. 89 (4), 1093–1101. doi: 10.1007/s00253-010-2935-2 20957359

[B13] Kumar S. Stecher G. Tamura K. (2016). MEGA7: molecular evolutionary genetics analysis version 7.0 for bigger datasets. Mol. Biol. Evol. 33 (7), 1870–1874. doi: 10.1093/molbev/msw054 27004904 PMC8210823

[B14] Kushmerick C. de Souza Castro M. Santos Cruz J. Bloch C. Beirão P. S. L. (1998). Functional and structural features of γ-zeathionins, a new class of sodium channel blockers. FEBS Lett. 440(3), 302–306. doi: 10.1016/S0014-5793(98)01480-X 9872391

[B15] Lescot M. Déhais P. Thijs G. Marchal K. Moreau Y. Van de Peer Y.. (2002). PlantCARE, a database of plant cis-acting regulatory elements and a portal to tools for in silico analysis of promoter sequences. Nucleic Acids Res. 30 (1), 325–327. doi: 10.1093/nar/30.1.325 11752327 PMC99092

[B16] Li R. An J.-p. You C.-x. Shu J. Wang X.-f. Hao Y.-j. (2018). Identification and expression of the CEP gene family in apple (Malus×domestica). J. Integr. Agric. 17 (2), 348–358. doi: 10.1016/s2095-3119(17)61653-8

[B17] Lima A. M. Azevedo M. I. G. Sousa L. M. Oliveira N. S. Andrade C. R. Freitas C. D. T.. (2022). Plant antimicrobial peptides: An overview about classification, toxicity and clinical applications. Int. J. Biol. Macromol 214, 10–21. doi: 10.1016/j.ijbiomac.2022.06.043 35700843

[B18] Livak K. J. Schmittgen T. D. (2001). Analysis of relative gene expression data using real-time quantitative PCR and the 2(-Delta Delta C(T)) Method. Methods 25 (4), 402–408. doi: 10.1006/meth.2001.1262 11846609

[B19] Mäser P. Thomine S. Schroeder J. I. Ward J. M. Hirschi K. Sze H.. (2001). Phylogenetic relationships within cation transporter families of arabidopsis. Plant Physiol. 126 (4), 1646–1667. doi: 10.1104/pp.126.4.1646 11500563 PMC117164

[B20] Mamidala P. Rajarapu S. P. Jones S. C. Mittapalli O. (2011). Identification and validation of reference genes for quantitative real-time polymerase chain reaction in Cimex lectularius. J. Med. Entomol 48 (4), 947–951. doi: 10.1603/me10262 21845960

[B21] Nguyen N. N. T. Lamotte O. Alsulaiman M. Ruffel S. Krouk G. Berger N.. (2023). Reduction in PLANT DEFENSIN 1 expression in Arabidopsis thaliana results in increased resistance to pathogens and zinc toxicity. J. Exp. Bot. 74(17): 5374–5393. doi: 10.1093/jxb/erad228 37326591

[B22] Parisi K. Shafee T. M. A. Quimbar P. van der Weerden N. L. Bleackley M. R. Anderson M. A. (2019). The evolution, function and mechanisms of action for plant defensins. Semin. Cell Dev. Biol. 88, 107–118. doi: 10.1016/j.semcdb.2018.02.004 29432955

[B23] Sadhu S. Jogam P. Gande K. Marapaka V. Penna S. Peddaboina V. (2023). Expression of radish defensin (RsAFP2) gene in chickpea (Cicer arietinum L.) confers resistance to Fusarium wilt disease. Mol. Biol. Rep. 50 (1), 11–18. doi: 10.1007/s11033-022-08021-9 36282461

[B24] Sagaram U. S. Pandurangi R. Kaur J. Smith T. J. Shah D. M. (2011). Structure-activity determinants in antifungal plant defensins MsDef1 and MtDef4 with different modes of action against Fusarium graminearum. PloS One 6 (4), e18550. doi: 10.1371/journal.pone.0018550 21533249 PMC3076432

[B25] Sathoff A. E. Velivelli S. L. S. Shah D. M. Samac D. A. (2019). Plant defensin peptides have antifungal and antibacterial activity against human and plant pathogens. Phytopathology 109 3, 402–408. doi: 10.1094/PHYTO-09-18-0331-R 30252607

[B26] Shahmiri M. Bleackley M. R. Dawson C. S. van der Weerden N. L. Anderson M. A. Mechler A. (2023). Membrane binding properties of plant defensins. Phytochemistry 209, 113618. doi: 10.1016/j.phytochem.2023.113618 36828099

[B27] Shahzad Z. Ranwez V. Fizames C. Marquès L. Le Martret B. Alassimone J.. (2013). Plant Defensin type 1 (PDF1): protein promiscuity and expression variation within the Arabidopsis genus shed light on zinc tolerance acquisition in Arabidopsis halleri. New Phytol. 200 (3), 820–833. doi: 10.1111/nph.12396 23865749

[B28] Terras F. R. Schoofs H. M. De Bolle M. F. Van Leuven F. Rees S. B. Vanderleyden J.. (1992). Analysis of two novel classes of plant antifungal proteins from radish (Raphanus sativus L.) seeds. J. Biol. Chem. 267 (22), 15301–15309. doi: 10.1016/s0021-9258(19)49534-3 1639777

[B29] Thomma B. P. Cammue B. P. Thevissen K. (2002). Plant defensins. Planta 216 (2), 193–202. doi: 10.1007/s00425-002-0902-6 12447532

[B30] Thompson J. D. Higgins D. G. Gibson T. J. (1994). CLUSTAL W: improving the sensitivity of progressive multiple sequence alignment through sequence weighting, position-specific gap penalties and weight matrix choice. Nucleic Acids Res. 22 (22), 4673–4680. doi: 10.1093/nar/22.22.4673 7984417 PMC308517

[B31] Trapnell C. Roberts A. Goff L. Pertea G. Kim D. Kelley D. R.. (2012). Differential gene and transcript expression analysis of RNA-seq experiments with TopHat and Cufflinks. Nat. Protoc. 7 (3), 562–578. doi: 10.1038/nprot.2012.016 22383036 PMC3334321

[B32] Vriens K. Cools T. L. Harvey P. J. Craik D. J. Braem A. Vleugels J.. (2016). The radish defensins RsAFP1 and RsAFP2 act synergistically with caspofungin against Candida albicans biofilms. Peptides 75, 71–79. doi: 10.1016/j.peptides.2015.11.001 26592804

[B33] Zheng X.-W. Deng-Xia Y. I. Lin-Hui S. Cong L. I. (2017). In silico genome-wide identification, phylogeny and expression analysis of the R2R3-MYB gene family in Medicago truncatula. J. Integr. Agric. 016 (007), 1576–1591. doi: 10.1016/S2095-3119(16)61521-6

