# Supplementary material for: Bioinformatic analysis of wheat defensin gene family and function verification of candidate genes
Source: Front Plant Sci. 2023 Oct 24;14:1279502. doi: 10.3389/fpls.2023.1279502 (PMC10628452; doi:10.3389/fpls.2023.1279502)
Supplement: Supplementary file 1 [file Table_1.docx]

Appendix S1 SRA database from NCBI

| **Name** | **Provider** | **Scientific Name** | **Variety** | **Age** | **Stress** | **Tissue** |
| --- | --- | --- | --- | --- | --- | --- |
| Salt_Root_of_Seeding_0 | SRR6367521 | Triticum aestivum | Kharchia Local | Seedling | salt | root |
| Salt_Leaf_of_Seeding_0 | SRR6367520 | Triticum aestivum | Kharchia Local | Seedling | salt | leaf |
| CK_Root_of_Seeding_0 | SRR6367519 | Triticum aestivum | Kharchia Local | Seedling | none | root |
| CK_Leaf_of_Seeding_0 | SRR6367518 | Triticum aestivum | Kharchia Local | Seedling | none | leaf |
| CK_Leaf_0 | SRR5747853 | Triticum aestivum | CB037 | 10-day-old | none | leaf |
| CK_Leaf_2 | SRR5747854 | Triticum aestivum | CB037 | 10-day-old | none | leaf |
| CK_Leaf_1 | SRR5747845 | Triticum aestivum | CB037 | 10-day-old | none | leaf |
| Drought_Leaf_2h_1 | SRR5747848 | Triticum aestivum | CB037 | 10-day-old | Drought_2h | leaf |
| Drought_Leaf_2h_0 | SRR5747843 | Triticum aestivum | CB037 | 10-day-old | Drought_2h | leaf |
| Drought_Leaf_2h_2 | SRR5747846 | Triticum aestivum | CB037 | 10-day-old | Drought_2h | leaf |
| Drought_Leaf_7h_1 | SRR5747859 | Triticum aestivum | CB037 | 10-day-old | Drought_7h | leaf |
| Drought_Leaf_7h_0 | SRR5747857 | Triticum aestivum | CB037 | 10-day-old | Drought_7h | leaf |
| Drought_Leaf_7h_2 | SRR5747847 | Triticum aestivum | CB037 | 10-day-old | Drought_7h | leaf |
| CK_Ear_of_GrainFormation_0 | SRR5383964 | Triticum aestivum | Luyuan502 | Grain formation | none | ear |
| CK_Ear_of_GrainFormation_1 | SRR5383963 | Triticum aestivum | Luyuan502 | Grain formation | none | ear |
| CK_Ear_of_GrainFormation_2 | SRR5383962 | Triticum aestivum | Luyuan502 | Grain formation | none | ear |
| Drough_Ear_of_GrainFormation_0 | SRR5383961 | Triticum aestivum | Luyuan502 | Grain formation | Drought | ear |
| Drough_Ear_of_GrainFormation_1 | SRR5383960 | Triticum aestivum | Luyuan502 | Grain formation | Drought | ear |
| Drough_Ear_of_GrainFormation_2 | SRR5383959 | Triticum aestivum | Luyuan502 | Grain formation | Drought | ear |
| CK_Ear_of_Flowering_0 | SRR5383957 | Triticum aestivum | Luyuan502 | Flowering stage | none | ear |
| CK_Ear_of_Flowering_2 | SRR5383955 | Triticum aestivum | Luyuan502 | Flowering stage | none | ear |
| CK_Ear_of_Flowering_1 | SRR5383954 | Triticum aestivum | Luyuan502 | Flowering stage | none | ear |
| Drough_Ear_of_Flowering_0 | SRR5383952 | Triticum aestivum | Luyuan502 | Flowering stage | Drought | ear |
| Drough_Ear_of_Flowering_1 | SRR5383950 | Triticum aestivum | Luyuan502 | Flowering stage | Drought | ear |
| Drough_Ear_of_Flowering_2 | SRR5383948 | Triticum aestivum | Luyuan502 | Flowering stage | Drought | ear |
| Drough_Leaf_of_Booting_0 | SRR5383947 | Triticum aestivum | Luyuan502 | Booting stage | Drought | leaf |
| Drough_Leaf_of_Booting_1 | SRR5383945 | Triticum aestivum | Luyuan502 | Booting stage | Drought | leaf |
| Drough_Leaf_of_Booting_2 | SRR5383944 | Triticum aestivum | Luyuan502 | Booting stage | Drought | leaf |
| Drough_Leaf_of_AntherDdifferentiation_0 | SRR5383935 | Triticum aestivum | Luyuan502 | Anther differentiation | Drought | leaf |
| Drough_Leaf_of_AntherDdifferentiation_2 | SRR5383934 | Triticum aestivum | Luyuan502 | Anther differentiation | Drought | leaf |
| Drough_Leaf_of_AntherDdifferentiation_1 | SRR5383933 | Triticum aestivum | Luyuan502 | Anther differentiation | Drought | leaf |
| NaHS_PEG_Leaf_24h_0 | SRR5317873 | Triticum aestivum | Luohan NO.6 | two weeks | 0.4 mM NaHS and 20% PEG6000 treatment for 24 h | leaf |
| NaHS_PEG_Leaf_24h_1 | SRR5317872 | Triticum aestivum | Luohan NO.6 | two weeks | 0.4 mM NaHS and 20% PEG6000 treatment for 25 h | leaf |
| PEG_Leaf_24h_0 | SRR5317871 | Triticum aestivum | Luohan NO.6 | two weeks | PEG6000 treatment for 24 h | leaf |
| PEG_Leaf_24h_1 | SRR5317870 | Triticum aestivum | Luohan NO.6 | two weeks | PEG6000 treatment for 25 h | leaf |
| NaHS_Leaf_24h_0 | SRR5317869 | Triticum aestivum | Luohan NO.6 | two weeks | 0.4 mM NaHS | leaf |
| NaHS_Leaf_24h_1 | SRR5317868 | Triticum aestivum | Luohan NO.6 | two weeks | 0.4 mM NaHS | leaf |
| CK_Leaf_24h_0 | SRR5317867 | Triticum aestivum | Luohan NO.6 | two weeks | none | leaf |
| CK_Leaf_24h_1 | SRR5317866 | Triticum aestivum | Luohan NO.6 | two weeks | none | leaf |
| CK_Leaf_0 | SRR4067818 | Triticum aestivum | Kharchia Local | Anthesis | none | Leaf |
| CK_Root_0 | SRR4085446 | Triticum aestivum | Kharchia Local | Anthesis | none | Leaf |
| Salt_Leaf_0 | SRR4085101 | Triticum aestivum | Kharchia Local | Anthesis | salt | Leaf |
| Salt_Root_0 | SRR4086039 | Triticum aestivum | Kharchia Local | Anthesis | salt | root |
| Na_48h_QM6_0 | SRR2306560 | Triticum aestivum | QM-6 | 1 week | 48 hour of salt stress | leaf |
| CK_48h_QM6_0 | SRR2306559 | Triticum aestivum | QM-6 | 1 week | 48 hour of ck | leaf |
| Na_24h_QM6_0 | SRR2306558 | Triticum aestivum | QM-6 | 1 week | 24 hour of salt stress | leaf |
| Na_12h_QM6_0 | SRR2306557 | Triticum aestivum | QM-6 | 1 week | 12 hour of salt stress | leaf |
| CK_24h_QM6_0 | SRR2306556 | Triticum aestivum | QM-6 | 1 week | 24 hour of ck | leaf |
| CK_12h_QM6_0 | SRR2306555 | Triticum aestivum | QM-6 | 1 week | 12 hour of ck | leaf |
| Na_6h_QM6_0 | SRR2306554 | Triticum aestivum | QM-6 | 1 week | 6 hour of salt stress | leaf |
| CK_6h_QM6_0 | SRR2306553 | Triticum aestivum | QM-6 | 1 week | 6 hour of ck | leaf |
| Na_48h_ChineseSpring_0 | SRR2306552 | Triticum aestivum | ChineseSpring | 1 week | 48 hour of salt stress | leaf |
| CK_48h_ChineseSpring_0 | SRR2306551 | Triticum aestivum | ChineseSpring | 1 week | 48 hour of ck | leaf |
| Na_24h_ChineseSpring_0 | SRR2306550 | Triticum aestivum | ChineseSpring | 1 week | 24 hour of salt stress | leaf |
| Na_12h_ChineseSpring_0 | SRR2306549 | Triticum aestivum | ChineseSpring | 1 week | 12 hour of salt stress | leaf |
| CK_24h_ChineseSpring_0 | SRR2306548 | Triticum aestivum | ChineseSpring | 1 week | 24 hour of ck | leaf |
| CK_12h_ChineseSpring_0 | SRR2306547 | Triticum aestivum | ChineseSpring | 1 week | 12 hour of ck | leaf |
| Na_6h_ChineseSpring_0 | SRR2306546 | Triticum aestivum | ChineseSpring | 1 week | 6 hour of salt stress | leaf |
| CK_6h_ChineseSpring_0 | SRR2306545 | Triticum aestivum | ChineseSpring | 1 week | 6 hour of ck | leaf |
| Heat_at_GrainFilling_HD29_0 | SRR1731532 | Triticum aestivum | HD29 | GrainFilling | Heat | leaf |
| Heat_at_GrainFilling_HD29_1 | SRR1731531 | Triticum aestivum | HD29 | GrainFilling | Heat | leaf |
| CK_at_GrainFilling_HD29_0 | SRR1728120 | Triticum aestivum | HD29 | GrainFilling | none | leaf |
| CK_at_GrainFilling_HD29_1 | SRR1728119 | Triticum aestivum | HD29 | GrainFilling | none | leaf |
| Heat_at_GrainFilling_HD85_1 | SRR1722393 | Triticum aestivum | HD85 | GrainFilling | Heat | leaf |
| Heat_at_GrainFilling_HD85_0 | SRR1722390 | Triticum aestivum | HD85 | GrainFilling | Heat | leaf |
| CK_at_GrainFilling_HD85_0 | SRR1714932 | Triticum aestivum | HD85 | GrainFilling | none | leaf |
| CK_at_GrainFilling_HD85_1 | SRR1714874 | Triticum aestivum | HD85 | GrainFilling | none | leaf |
| Heat_Drought_6h_1 | SRR1542417 | Triticum aestivum | TAM107 | 1 week | 6 hour of drought&heat combined stress | leaf |
| Heat_Drought_6h_0 | SRR1542416 | Triticum aestivum | TAM107 | 1 week | 6 hour of drought&heat combined stress | leaf |
| Heat_Drought_1h_0 | SRR1542415 | Triticum aestivum | TAM107 | 1 week | 1 hour of drought&heat combined stress | leaf |
| Heat_Drought_1h_1 | SRR1542414 | Triticum aestivum | TAM107 | 1 week | 1 hour of drought&heat combined stress | leaf |
| Heat_6h_0 | SRR1542413 | Triticum aestivum | TAM107 | 1 week | 6 hour of heat stress | leaf |
| Heat_6h_1 | SRR1542412 | Triticum aestivum | TAM107 | 1 week | 6 hour of heat stress | leaf |
| Heat_1h_0 | SRR1542411 | Triticum aestivum | TAM107 | 1 week | 1 hour of heat stress | leaf |
| Heat_1h_1 | SRR1542410 | Triticum aestivum | TAM107 | 1 week | 1 hour of heat stress | leaf |
| Drought_6h_0 | SRR1542409 | Triticum aestivum | TAM107 | 1 week | 6 hour of drought stress | leaf |
| Drought_6h_1 | SRR1542408 | Triticum aestivum | TAM107 | 1 week | 6 hour of drought stress | leaf |
| Drought_1h_1 | SRR1542407 | Triticum aestivum | TAM107 | 1 week | 1 hour of drought stress | leaf |
| Drought_1h_0 | SRR1542406 | Triticum aestivum | TAM107 | 1 week | 1 hour of drought stress | leaf |
| CK1_0 | SRR1542405 | Triticum aestivum | TAM107 | 1 week | ck | leaf |
| CK1_1 | SRR1542404 | Triticum aestivum | TAM107 | 1 week | ck | leaf |
| ck_6h_spikelets_anthesis_NIL51_1 | SRA298 | Triticum aestivum | NIL51 from BC55F2 Remus in CM-82036 | anthesis | mock inoculation 6 hours | spikelets (only using palea, lemma and rachis) |
| ck_6h_spikelets_anthesis_NIL51_2 | SRA299 | Triticum aestivum | NIL51 from BC55F2 Remus in CM-82036 | anthesis | mock inoculation 6 hours | spikelets (only using palea, lemma and rachis) |
| ck_6h_spikelets_anthesis_NIL51_3 | SRA300 | Triticum aestivum | NIL51 from BC55F2 Remus in CM-82036 | anthesis | mock inoculation 6 hours | spikelets (only using palea, lemma and rachis) |
| ck_48h_spikelets_anthesis_NIL51_1 | SRA301 | Triticum aestivum | NIL51 from BC55F2 Remus in CM-82036 | anthesis | mock inoculation 48 hours | spikelets (only using palea, lemma and rachis) |
| ck_48h_spikelets_anthesis_NIL51_2 | SRA302 | Triticum aestivum | NIL51 from BC55F2 Remus in CM-82036 | anthesis | mock inoculation 48 hours | spikelets (only using palea, lemma and rachis) |
| ck_48h_spikelets_anthesis_NIL51_3 | SRA303 | Triticum aestivum | NIL51 from BC55F2 Remus in CM-82036 | anthesis | mock inoculation 48 hours | spikelets (only using palea, lemma and rachis) |
| ck_3h_spikelets_anthesis_NIL51_1 | SRA304 | Triticum aestivum | NIL51 from BC55F2 Remus in CM-82036 | anthesis | mock inoculation 3 hours | spikelets (only using palea, lemma and rachis) |
| ck_3h_spikelets_anthesis_NIL51_2 | SRA305 | Triticum aestivum | NIL51 from BC55F2 Remus in CM-82036 | anthesis | mock inoculation 3 hours | spikelets (only using palea, lemma and rachis) |
| ck_3h_spikelets_anthesis_NIL51_3 | SRA306 | Triticum aestivum | NIL51 from BC55F2 Remus in CM-82036 | anthesis | mock inoculation 3 hours | spikelets (only using palea, lemma and rachis) |
| ck_36h_spikelets_anthesis_NIL51_1 | SRA307 | Triticum aestivum | NIL51 from BC55F2 Remus in CM-82036 | anthesis | mock inoculation 36 hours | spikelets (only using palea, lemma and rachis) |
| ck_36h_spikelets_anthesis_NIL51_2 | SRA308 | Triticum aestivum | NIL51 from BC55F2 Remus in CM-82036 | anthesis | mock inoculation 36 hours | spikelets (only using palea, lemma and rachis) |
| ck_36h_spikelets_anthesis_NIL51_3 | SRA309 | Triticum aestivum | NIL51 from BC55F2 Remus in CM-82036 | anthesis | mock inoculation 36 hours | spikelets (only using palea, lemma and rachis) |
| ck_24h_spikelets_anthesis_NIL51_1 | SRA310 | Triticum aestivum | NIL51 from BC55F2 Remus in CM-82036 | anthesis | mock inoculation 24 hours | spikelets (only using palea, lemma and rachis) |
| ck_24h_spikelets_anthesis_NIL51_2 | SRA311 | Triticum aestivum | NIL51 from BC55F2 Remus in CM-82036 | anthesis | mock inoculation 24 hours | spikelets (only using palea, lemma and rachis) |
| ck_24h_spikelets_anthesis_NIL51_3 | SRA312 | Triticum aestivum | NIL51 from BC55F2 Remus in CM-82036 | anthesis | mock inoculation 24 hours | spikelets (only using palea, lemma and rachis) |
| ck_12h_spikelets_anthesis_NIL51_1 | SRA313 | Triticum aestivum | NIL51 from BC55F2 Remus in CM-82036 | anthesis | mock inoculation 12 hours | spikelets (only using palea, lemma and rachis) |
| ck_12h_spikelets_anthesis_NIL51_2 | SRA314 | Triticum aestivum | NIL51 from BC55F2 Remus in CM-82036 | anthesis | mock inoculation 12 hours | spikelets (only using palea, lemma and rachis) |
| ck_12h_spikelets_anthesis_NIL51_3 | SRA315 | Triticum aestivum | NIL51 from BC55F2 Remus in CM-82036 | anthesis | mock inoculation 12 hours | spikelets (only using palea, lemma and rachis) |
| Fusarium graminearum_6h_spikelets_anthesis_NIL51_1 | SRA316 | Triticum aestivum | NIL51 from BC55F2 Remus in CM-82036 | anthesis | Fusarium graminearum inoculation 6 hours | spikelets (only using palea, lemma and rachis) |
| Fusarium graminearum_6h_spikelets_anthesis_NIL51_2 | SRA317 | Triticum aestivum | NIL51 from BC55F2 Remus in CM-82036 | anthesis | Fusarium graminearum inoculation 6 hours | spikelets (only using palea, lemma and rachis) |
| Fusarium graminearum_6h_spikelets_anthesis_NIL51_3 | SRA318 | Triticum aestivum | NIL51 from BC55F2 Remus in CM-82036 | anthesis | Fusarium graminearum inoculation 6 hours | spikelets (only using palea, lemma and rachis) |
| Fusarium graminearum_48h_spikelets_anthesis_NIL51_1 | SRA319 | Triticum aestivum | NIL51 from BC55F2 Remus in CM-82036 | anthesis | Fusarium graminearum inoculation 48 hours | spikelets (only using palea, lemma and rachis) |
| Fusarium graminearum_48h_spikelets_anthesis_NIL51_2 | SRA320 | Triticum aestivum | NIL51 from BC55F2 Remus in CM-82036 | anthesis | Fusarium graminearum inoculation 48 hours | spikelets (only using palea, lemma and rachis) |
| Fusarium graminearum_48h_spikelets_anthesis_NIL51_3 | SRA321 | Triticum aestivum | NIL51 from BC55F2 Remus in CM-82036 | anthesis | Fusarium graminearum inoculation 48 hours | spikelets (only using palea, lemma and rachis) |
| Fusarium graminearum_3h_spikelets_anthesis_NIL51_1 | SRA322 | Triticum aestivum | NIL51 from BC55F2 Remus in CM-82036 | anthesis | Fusarium graminearum inoculation 3 hours | spikelets (only using palea, lemma and rachis) |
| Fusarium graminearum_3h_spikelets_anthesis_NIL51_2 | SRA323 | Triticum aestivum | NIL51 from BC55F2 Remus in CM-82036 | anthesis | Fusarium graminearum inoculation 3 hours | spikelets (only using palea, lemma and rachis) |
| Fusarium graminearum_3h_spikelets_anthesis_NIL51_3 | SRA324 | Triticum aestivum | NIL51 from BC55F2 Remus in CM-82036 | anthesis | Fusarium graminearum inoculation 3 hours | spikelets (only using palea, lemma and rachis) |
| Fusarium graminearum_36h_spikelets_anthesis_NIL51_1 | SRA325 | Triticum aestivum | NIL51 from BC55F2 Remus in CM-82036 | anthesis | Fusarium graminearum inoculation 36 hours | spikelets (only using palea, lemma and rachis) |
| Fusarium graminearum_36h_spikelets_anthesis_NIL51_2 | SRA326 | Triticum aestivum | NIL51 from BC55F2 Remus in CM-82036 | anthesis | Fusarium graminearum inoculation 36 hours | spikelets (only using palea, lemma and rachis) |
| Fusarium graminearum_36h_spikelets_anthesis_NIL51_3 | SRA327 | Triticum aestivum | NIL51 from BC55F2 Remus in CM-82036 | anthesis | Fusarium graminearum inoculation 36 hours | spikelets (only using palea, lemma and rachis) |
| Fusarium graminearum_24h_spikelets_anthesis_NIL51_1 | SRA328 | Triticum aestivum | NIL51 from BC55F2 Remus in CM-82036 | anthesis | Fusarium graminearum inoculation 24 hours | spikelets (only using palea, lemma and rachis) |
| Fusarium graminearum_24h_spikelets_anthesis_NIL51_2 | SRA329 | Triticum aestivum | NIL51 from BC55F2 Remus in CM-82036 | anthesis | Fusarium graminearum inoculation 24 hours | spikelets (only using palea, lemma and rachis) |
| Fusarium graminearum_24h_spikelets_anthesis_NIL51_3 | SRA330 | Triticum aestivum | NIL51 from BC55F2 Remus in CM-82036 | anthesis | Fusarium graminearum inoculation 24 hours | spikelets (only using palea, lemma and rachis) |
| Fusarium graminearum_12h_spikelets_anthesis_NIL51_1 | SRA331 | Triticum aestivum | NIL51 from BC55F2 Remus in CM-82036 | anthesis | Fusarium graminearum inoculation 12 hours | spikelets (only using palea, lemma and rachis) |
| Fusarium graminearum_12h_spikelets_anthesis_NIL51_2 | SRA332 | Triticum aestivum | NIL51 from BC55F2 Remus in CM-82036 | anthesis | Fusarium graminearum inoculation 12 hours | spikelets (only using palea, lemma and rachis) |
| Fusarium graminearum_12h_spikelets_anthesis_NIL51_3 | SRA333 | Triticum aestivum | NIL51 from BC55F2 Remus in CM-82036 | anthesis | Fusarium graminearum inoculation 12 hours | spikelets (only using palea, lemma and rachis) |
| ck_6h_spikelets_anthesis_NIL38_1 | SRA334 | Triticum aestivum | NIL38 from BC55F2 Remus in CM-82036 | anthesis | mock inoculation 6 hours | spikelets (only using palea, lemma and rachis) |
| ck_6h_spikelets_anthesis_NIL38_2 | SRA335 | Triticum aestivum | NIL38 from BC55F2 Remus in CM-82036 | anthesis | mock inoculation 6 hours | spikelets (only using palea, lemma and rachis) |
| ck_6h_spikelets_anthesis_NIL38_3 | SRA336 | Triticum aestivum | NIL38 from BC55F2 Remus in CM-82036 | anthesis | mock inoculation 6 hours | spikelets (only using palea, lemma and rachis) |
| ck_48h_spikelets_anthesis_NIL38_1 | SRA337 | Triticum aestivum | NIL38 from BC55F2 Remus in CM-82036 | anthesis | mock inoculation 48 hours | spikelets (only using palea, lemma and rachis) |
| ck_48h_spikelets_anthesis_NIL38_2 | SRA338 | Triticum aestivum | NIL38 from BC55F2 Remus in CM-82036 | anthesis | mock inoculation 48 hours | spikelets (only using palea, lemma and rachis) |
| ck_48h_spikelets_anthesis_NIL38_3 | SRA339 | Triticum aestivum | NIL38 from BC55F2 Remus in CM-82036 | anthesis | mock inoculation 48 hours | spikelets (only using palea, lemma and rachis) |
| ck_3h_spikelets_anthesis_NIL38_1 | SRA340 | Triticum aestivum | NIL38 from BC55F2 Remus in CM-82036 | anthesis | mock inoculation 3 hours | spikelets (only using palea, lemma and rachis) |
| ck_3h_spikelets_anthesis_NIL38_2 | SRA341 | Triticum aestivum | NIL38 from BC55F2 Remus in CM-82036 | anthesis | mock inoculation 3 hours | spikelets (only using palea, lemma and rachis) |
| ck_3h_spikelets_anthesis_NIL38_3 | SRA342 | Triticum aestivum | NIL38 from BC55F2 Remus in CM-82036 | anthesis | mock inoculation 3 hours | spikelets (only using palea, lemma and rachis) |
| ck_36h_spikelets_anthesis_NIL38_1 | SRA343 | Triticum aestivum | NIL38 from BC55F2 Remus in CM-82036 | anthesis | mock inoculation 36 hours | spikelets (only using palea, lemma and rachis) |
| ck_36h_spikelets_anthesis_NIL38_2 | SRA344 | Triticum aestivum | NIL38 from BC55F2 Remus in CM-82036 | anthesis | mock inoculation 36 hours | spikelets (only using palea, lemma and rachis) |
| ck_36h_spikelets_anthesis_NIL38_3 | SRA345 | Triticum aestivum | NIL38 from BC55F2 Remus in CM-82036 | anthesis | mock inoculation 36 hours | spikelets (only using palea, lemma and rachis) |
| ck_24h_spikelets_anthesis_NIL38_1 | SRA346 | Triticum aestivum | NIL38 from BC55F2 Remus in CM-82036 | anthesis | mock inoculation 24 hours | spikelets (only using palea, lemma and rachis) |
| ck_24h_spikelets_anthesis_NIL38_2 | SRA347 | Triticum aestivum | NIL38 from BC55F2 Remus in CM-82036 | anthesis | mock inoculation 24 hours | spikelets (only using palea, lemma and rachis) |
| ck_24h_spikelets_anthesis_NIL38_3 | SRA348 | Triticum aestivum | NIL38 from BC55F2 Remus in CM-82036 | anthesis | mock inoculation 24 hours | spikelets (only using palea, lemma and rachis) |
| ck_12h_spikelets_anthesis_NIL38_1 | SRA349 | Triticum aestivum | NIL38 from BC55F2 Remus in CM-82036 | anthesis | mock inoculation 12 hours | spikelets (only using palea, lemma and rachis) |
| ck_12h_spikelets_anthesis_NIL38_2 | SRA350 | Triticum aestivum | NIL38 from BC55F2 Remus in CM-82036 | anthesis | mock inoculation 12 hours | spikelets (only using palea, lemma and rachis) |
| ck_12h_spikelets_anthesis_NIL38_3 | SRA351 | Triticum aestivum | NIL38 from BC55F2 Remus in CM-82036 | anthesis | mock inoculation 12 hours | spikelets (only using palea, lemma and rachis) |
| Fusarium graminearum_6h_spikelets_anthesis_NIL38_1 | SRA352 | Triticum aestivum | NIL38 from BC55F2 Remus in CM-82036 | anthesis | Fusarium graminearum inoculation 6 hours | spikelets (only using palea, lemma and rachis) |
| Fusarium graminearum_6h_spikelets_anthesis_NIL38_2 | SRA353 | Triticum aestivum | NIL38 from BC55F2 Remus in CM-82036 | anthesis | Fusarium graminearum inoculation 6 hours | spikelets (only using palea, lemma and rachis) |
| Fusarium graminearum_6h_spikelets_anthesis_NIL38_3 | SRA354 | Triticum aestivum | NIL38 from BC55F2 Remus in CM-82036 | anthesis | Fusarium graminearum inoculation 6 hours | spikelets (only using palea, lemma and rachis) |
| Fusarium graminearum_48h_spikelets_anthesis_NIL38_1 | SRA355 | Triticum aestivum | NIL38 from BC55F2 Remus in CM-82036 | anthesis | Fusarium graminearum inoculation 48 hours | spikelets (only using palea, lemma and rachis) |
| Fusarium graminearum_48h_spikelets_anthesis_NIL38_2 | SRA356 | Triticum aestivum | NIL38 from BC55F2 Remus in CM-82036 | anthesis | Fusarium graminearum inoculation 48 hours | spikelets (only using palea, lemma and rachis) |
| Fusarium graminearum_48h_spikelets_anthesis_NIL38_3 | SRA357 | Triticum aestivum | NIL38 from BC55F2 Remus in CM-82036 | anthesis | Fusarium graminearum inoculation 48 hours | spikelets (only using palea, lemma and rachis) |
| Fusarium graminearum_3h_spikelets_anthesis_NIL38_1 | SRA358 | Triticum aestivum | NIL38 from BC55F2 Remus in CM-82036 | anthesis | Fusarium graminearum inoculation 3 hours | spikelets (only using palea, lemma and rachis) |
| Fusarium graminearum_3h_spikelets_anthesis_NIL38_2 | SRA359 | Triticum aestivum | NIL38 from BC55F2 Remus in CM-82036 | anthesis | Fusarium graminearum inoculation 3 hours | spikelets (only using palea, lemma and rachis) |
| Fusarium graminearum_3h_spikelets_anthesis_NIL38_3 | SRA360 | Triticum aestivum | NIL38 from BC55F2 Remus in CM-82036 | anthesis | Fusarium graminearum inoculation 3 hours | spikelets (only using palea, lemma and rachis) |
| Fusarium graminearum_36h_spikelets_anthesis_NIL38_1 | SRA361 | Triticum aestivum | NIL38 from BC55F2 Remus in CM-82036 | anthesis | Fusarium graminearum inoculation 36 hours | spikelets (only using palea, lemma and rachis) |
| Fusarium graminearum_36h_spikelets_anthesis_NIL38_3 | SRA362 | Triticum aestivum | NIL38 from BC55F2 Remus in CM-82036 | anthesis | Fusarium graminearum inoculation 36 hours | spikelets (only using palea, lemma and rachis) |
| Fusarium graminearum_24h_spikelets_anthesis_NIL38_1 | SRA363 | Triticum aestivum | NIL38 from BC55F2 Remus in CM-82036 | anthesis | Fusarium graminearum inoculation 36 hours | spikelets (only using palea, lemma and rachis) |
| Fusarium graminearum_24h_spikelets_anthesis_NIL38_2 | SRA364 | Triticum aestivum | NIL38 from BC55F2 Remus in CM-82036 | anthesis | Fusarium graminearum inoculation 24 hours | spikelets (only using palea, lemma and rachis) |
| Fusarium graminearum_24h_spikelets_anthesis_NIL38_3 | SRA365 | Triticum aestivum | NIL38 from BC55F2 Remus in CM-82036 | anthesis | Fusarium graminearum inoculation 24 hours | spikelets (only using palea, lemma and rachis) |
| Fusarium graminearum_12h_spikelets_anthesis_NIL38_1 | SRA366 | Triticum aestivum | NIL38 from BC55F2 Remus in CM-82036 | anthesis | Fusarium graminearum inoculation 24 hours | spikelets (only using palea, lemma and rachis) |
| Fusarium graminearum_12h_spikelets_anthesis_NIL38_2 | SRA367 | Triticum aestivum | NIL38 from BC55F2 Remus in CM-82036 | anthesis | Fusarium graminearum inoculation 12 hours | spikelets (only using palea, lemma and rachis) |
| Fusarium graminearum_12h_spikelets_anthesis_NIL38_3 | SRA368 | Triticum aestivum | NIL38 from BC55F2 Remus in CM-82036 | anthesis | Fusarium graminearum inoculation 12 hours | spikelets (only using palea, lemma and rachis) |
| ck_Vuka_leaf_1 | SRA369 | Triticum aestivum | NIL38 from BC55F2 Remus in CM-82036 | anthesis | Fusarium graminearum inoculation 12 hours | spikelets (only using palea, lemma and rachis) |
| ck_Vuka_leaf_2 | SRA542 | Triticum aestivum | Vuka | three leaf stage | control | leaf |
| ck_Vuka_leaf_3 | SRA543 | Triticum aestivum | Vuka | three leaf stage | control | leaf |
| stripe rust 87/66 1d_Vuka_leaf_2 | SRA544 | Triticum aestivum | Vuka | three leaf stage | control | leaf |
| stripe rust 87/66 1d_Vuka_leaf_3 | SRA545 | Triticum aestivum | Vuka | three leaf stage | stripe rust pathogen 87/66 1 day | leaf |
| stripe rust 87/66 2d_Vuka_leaf_1 | SRA546 | Triticum aestivum | Vuka | three leaf stage | stripe rust pathogen 87/66 1 day | leaf |
| stripe rust 87/66 2d_Vuka_leaf_2 | SRA547 | Triticum aestivum | Vuka | three leaf stage | stripe rust pathogen 87/66 1 day | leaf |
| stripe rust 87/66 2d_Vuka_leaf_3 | SRA548 | Triticum aestivum | Vuka | three leaf stage | stripe rust pathogen 87/66 2 days | leaf |
| stripe rust 87/66 3d_Vuka_leaf_1 | SRA549 | Triticum aestivum | Vuka | three leaf stage | stripe rust pathogen 87/66 2 days | leaf |
| stripe rust 87/66 3d_Vuka_leaf_2 | SRA550 | Triticum aestivum | Vuka | three leaf stage | stripe rust pathogen 87/66 2 days | leaf |
| stripe rust 87/66 3d_Vuka_leaf_3 | SRA551 | Triticum aestivum | Vuka | three leaf stage | stripe rust pathogen 87/66 3 days | leaf |
| stripe rust 87/66 5d_Vuka_leaf_1 | SRA552 | Triticum aestivum | Vuka | three leaf stage | stripe rust pathogen 87/66 3 days | leaf |
| stripe rust 87/66 5d_Vuka_leaf_2 | SRA553 | Triticum aestivum | Vuka | three leaf stage | stripe rust pathogen 87/66 3 days | leaf |
| stripe rust 87/66 7d_Vuka_leaf_1 | SRA554 | Triticum aestivum | Vuka | three leaf stage | stripe rust pathogen 87/66 5 days | leaf |
| stripe rust 87/66 7d_Vuka_leaf_2 | SRA555 | Triticum aestivum | Vuka | three leaf stage | stripe rust pathogen 87/66 5 days | leaf |
| stripe rust 87/66 7d_Vuka_leaf_3 | SRA556 | Triticum aestivum | Vuka | three leaf stage | stripe rust pathogen 87/66 5 days | leaf |
| stripe rust 87/66 9d_Vuka_leaf_1 | SRA557 | Triticum aestivum | Vuka | three leaf stage | stripe rust pathogen 87/66 7 days | leaf |
| stripe rust 87/66 9d_Vuka_leaf_2 | SRA558 | Triticum aestivum | Vuka | three leaf stage | stripe rust pathogen 87/66 7 days | leaf |
| stripe rust 87/66 9d_Vuka_leaf_3 | SRA559 | Triticum aestivum | Vuka | three leaf stage | stripe rust pathogen 87/66 7 days | leaf |
| stripe rust 87/66 11d_Vuka_leaf_1 | SRA560 | Triticum aestivum | Vuka | three leaf stage | stripe rust pathogen 87/66 9 days | leaf |
| stripe rust 87/66 11d_Vuka_leaf_2 | SRA561 | Triticum aestivum | Vuka | three leaf stage | stripe rust pathogen 87/66 9 days | leaf |
| stripe rust 87/66 11d_Vuka_leaf_3 | SRA562 | Triticum aestivum | Vuka | three leaf stage | stripe rust pathogen 87/66 9 days | leaf |
| ck_Avocet+Yr5_leaf_1 | SRA563 | Triticum aestivum | Vuka | three leaf stage | stripe rust pathogen 87/66 11 days | leaf |
| ck_Avocet+Yr5_leaf_3 | SRA564 | Triticum aestivum | Vuka | three leaf stage | stripe rust pathogen 87/66 11 days | leaf |
| stripe rust 87/66 1d_Avocet+Yr5_leaf_1 | SRA565 | Triticum aestivum | Vuka | three leaf stage | stripe rust pathogen 87/66 11 days | leaf |
| stripe rust 87/66 1d_Avocet+Yr5_leaf_2 | SRA566 | Triticum aestivum | Avocet+Yr5 | three leaf stage | control | leaf |
| stripe rust 87/66 1d_Avocet+Yr5_leaf_3 | SRA567 | Triticum aestivum | Avocet+Yr5 | three leaf stage | control | leaf |
| stripe rust 87/66 2d_Avocet+Yr5_leaf_1 | SRA568 | Triticum aestivum | Avocet+Yr5 | three leaf stage | control | leaf |
| stripe rust 87/66 2d_Avocet+Yr5_leaf_2 | SRA569 | Triticum aestivum | Avocet+Yr5 | three leaf stage | stripe rust pathogen 87/66 1 day | leaf |
| stripe rust 87/66 2d_Avocet+Yr5_leaf_3 | SRA570 | Triticum aestivum | Avocet+Yr5 | three leaf stage | stripe rust pathogen 87/66 1 day | leaf |
| stripe rust 87/66 3d_Avocet+Yr5_leaf_1 | SRA571 | Triticum aestivum | Avocet+Yr5 | three leaf stage | stripe rust pathogen 87/66 1 day | leaf |
| stripe rust 87/66 3d_Avocet+Yr5_leaf_2 | SRA572 | Triticum aestivum | Avocet+Yr5 | three leaf stage | stripe rust pathogen 87/66 2 days | leaf |
| stripe rust 87/66 3d_Avocet+Yr5_leaf_3 | SRA573 | Triticum aestivum | Avocet+Yr5 | three leaf stage | stripe rust pathogen 87/66 2 days | leaf |
| stripe rust 87/66 5d_Avocet+Yr5_leaf_2 | SRA574 | Triticum aestivum | Avocet+Yr5 | three leaf stage | stripe rust pathogen 87/66 2 days | leaf |
| stripe rust 87/66 5d_Avocet+Yr5_leaf_3 | SRA575 | Triticum aestivum | Avocet+Yr5 | three leaf stage | stripe rust pathogen 87/66 3 days | leaf |
| Magnaporthe oryzae_Bangladesh_leaf_2_1 | SRA576 | Triticum aestivum | Avocet+Yr5 | three leaf stage | stripe rust pathogen 87/66 3 days | leaf |
| Magnaporthe oryzae_Bangladesh_leaf_2_2 | SRA577 | Triticum aestivum | Avocet+Yr5 | three leaf stage | stripe rust pathogen 87/66 3 days | leaf |
| Magnaporthe oryzae_Bangladesh_leaf_12_1 | SRA578 | Triticum aestivum | Avocet+Yr5 | three leaf stage | stripe rust pathogen 87/66 5 days | leaf |
| Magnaporthe oryzae_Bangladesh_leaf_12_2 | SRA579 | Triticum aestivum | Avocet+Yr5 | three leaf stage | stripe rust pathogen 87/66 5 days | leaf |
| Magnaporthe oryzae_Bangladesh_leaf_7_1 | SRA580 | Triticum aestivum | Avocet+Yr5 | three leaf stage | stripe rust pathogen 87/66 5 days | leaf |
| Magnaporthe oryzae_Bangladesh_leaf_7_2 | SRA378 | Triticum aestivum | unknown (Bangladesh) | grain filling | Magnaporthe oryzae symptomatic | leaf |
| Magnaporthe oryzae_Bangladesh_leaf_5_1 | SRA379 | Triticum aestivum | unknown (Bangladesh) | grain filling | Magnaporthe oryzae symptomatic | leaf |
| Magnaporthe oryzae_Bangladesh_leaf_5_2 | SRA380 | Triticum aestivum | unknown (Bangladesh) | grain filling | Magnaporthe oryzae symptomatic | leaf |
| ck_Bangladesh_leaf_2_2 | SRA381 | Triticum aestivum | unknown (Bangladesh) | grain filling | Magnaporthe oryzae symptomatic | leaf |
| ck_Bangladesh_leaf_12_1 | SRA382 | Triticum aestivum | unknown (Bangladesh) | grain filling | Magnaporthe oryzae symptomatic | leaf |
| ck_Bangladesh_leaf_12_2 | SRA383 | Triticum aestivum | unknown (Bangladesh) | grain filling | Magnaporthe oryzae symptomatic | leaf |
| ck_Bangladesh_leaf_5_1 | SRA384 | Triticum aestivum | unknown (Bangladesh) | grain filling | Magnaporthe oryzae symptomatic | leaf |
| ck_Bangladesh_leaf_5_2 | SRA385 | Triticum aestivum | unknown (Bangladesh) | grain filling | Magnaporthe oryzae symptomatic | leaf |
| ck_Bangladesh_leaf_7_1 | SRA386 | Triticum aestivum | unknown (Bangladesh) | grain filling | Magnaporthe oryzae asymptomatic | leaf |
| ck_Bangladesh_leaf_7_2 | SRA387 | Triticum aestivum | unknown (Bangladesh) | grain filling | Magnaporthe oryzae asymptomatic | leaf |
| grain_Chinese Spring | SRA388 | Triticum aestivum | unknown (Bangladesh) | grain filling | Magnaporthe oryzae asymptomatic | leaf |
| spike_Chinese Spring | SRA389 | Triticum aestivum | unknown (Bangladesh) | grain filling | Magnaporthe oryzae asymptomatic | leaf |
| stem_Chinese Spring | SRA390 | Triticum aestivum | unknown (Bangladesh) | grain filling | Magnaporthe oryzae asymptomatic | leaf |
| roots_Chinese Spring | SRA391 | Triticum aestivum | unknown (Bangladesh) | grain filling | Magnaporthe oryzae asymptomatic | leaf |
| seedling_Chinese Spring | SRA392 | Triticum aestivum | unknown (Bangladesh) | grain filling | Magnaporthe oryzae asymptomatic | leaf |
