# Supplementary material for: Bioinformatic analysis of wheat defensin gene family and function verification of candidate genes
Source: Front Plant Sci. 2023 Oct 24;14:1279502. doi: 10.3389/fpls.2023.1279502 (PMC10628452; doi:10.3389/fpls.2023.1279502)
Supplement: Supplementary file 2 [file Table_2.docx]

Appendix S2 Primer of *TaPDF* genes

| Primer name | sequences（5' to 3'） |
| --- | --- |
| TaPDF2.23 249 XhoI Part27-F | TTTGGAGAGGACACGCTCGAGATGGCGCCCTCTCGTCGC |
| TaPDF2.23 249 XhoI Part27-R | GCCCTTGCTCACCATCTCGAGGCAGGCCCTCTTGCAG |
| TaPDF4.9 228 XhoI pART27-F | TTTGGAGAGGACACGCTCGAGATGGAGTCATCACACAAG |
| TaPDF4.9 228 XhoI pART27-R | GCCCTTGCTCACCATCTCGAGGCACTCCTTCGTGCAC |
| Ta2291F | GCTCTCCAACAACATTGCCAAC |
| Ta2291R | GCTTCTGCCTGTCACATACGC |
| TaPDF2.12F | ATGGCCGCCGCGCCCGC |
| TaPDF2.12R | GCAGGTGCGCTTGCAGAAG |
| TaPDF2.23F | ATGGCGCCCTCTCGTCGC |
| TaPDF2.23R | GCAGGCCCTCTTGCAG |
| TaPDF2.15F | ATGAATTCATCCCGCAAG |
| TaPDF2.15R | GCATGGTGTTTTGCACATG |
| TaPDF2.20F | ATGGCGTCCCCCCGTCC |
| TaPDF2.20R | GCAGTCCCGCTCGCAG |
| TaPDF4.9F | ATGGAGTCATCACACAAG |
| TaPDF4.9R | GCACTCCTTCGTGCAC |
| TaPDF5.4F | ATGGAGGCTTCACGCAG |
| TaPDF5.4R | ATGGTGGCAGTGGGTGGTG |
